# α-Synuclein aggregates amplified from patient-derived Lewy bodies recapitulate Lewy body diseases in mice

**DOI:** 10.1038/s41467-023-42705-5

**Published:** 2023-10-28

**Authors:** Norihito Uemura, Nicholas P. Marotta, Jahan Ara, Emily S. Meymand, Bin Zhang, Hiroshi Kameda, Masato Koike, Kelvin C. Luk, John Q. Trojanowski, Virginia M.-Y. Lee

**Affiliations:** 1 Department of Pathology and Laboratory Medicine, Institute on Aging and Center for Neurodegenerative Disease Research, University of Pennsylvania School of Medicine, Philadelphia, PA 19104-2676 USA; 2 Department of Cell Biology and Neuroscience, Juntendo University Graduate School of Medicine, Tokyo, Japan

**Keywords:** Parkinson's disease, Neurodegeneration, Cell death in the nervous system

## Abstract

Extraction of α-Synuclein (αSyn) aggregates from Lewy body disease (LBD) brains has been widely described yet templated fibrillization of LB-αSyn often fails to propagate its structural and functional properties. We recently demonstrated that aggregates amplified from LB-αSyn (ampLB) show distinct biological activities in vitro compared to human αSyn preformed fibrils (hPFF) formed de novo. Here we compare the in vivo biological activities of hPFF and ampLB regarding seeding activity, latency in inducing pathology, distribution of pathology, inclusion morphology, and cell-type preference. Injection of ampLB into mice expressing only human αSyn (male Thy1:*SNCA*/*Snca*^–/–^ mice) induced pathologies similar to those of LBD subjects that were distinct from those induced by hPFF-injection or developing spontaneously with aging. Importantly, αSyn aggregates in ampLB-injected Thy1:*SNCA*/*Snca*^–/–^ mice maintained the unique biological and conformational features of original LB-αSyn. These results indicate that ampLB-injection, rather than conventional PFF-injection or αSyn overexpression, faithfully models key aspects of LBD.

## Abstract (web summary)

α-Synuclein aggregates in Lewy bodies (LBs) have not been widely used for research due to the limited availability of diseased brains. Here, the authors report a mouse model that recapitulates LB diseases using the LB amplification method.

## Introduction

Lewy bodies (LB) are intraneuronal inclusions largely composed of α-Synuclein (αSyn) and are found in Lewy body diseases (LBD), a family that encompasses Parkinson’s disease (PD), dementia with Lewy bodies (DLB), and Alzheimer’s disease (AD) with LB co-pathology^1,2^. Patients with PD first show motor dysfunction (i.e., parkinsonism) but eventually develop cognitive dysfunction, diagnosed as PD with dementia (PDD)^3^. A diagnosis of DLB is given to patients in whom cognitive dysfunction occurs before or concurrently with the motor dysfunction^4^. LB co-pathology is also commonly observed in subjects pathologically diagnosed with AD^2,5^. An additional synucleinopathy is represented by multiple system atrophy (MSA), a pathologically distinct entity characterized by the abundance of oligodendroglial αSyn pathology with rare neuronal inclusions^6,7^.

αSyn is highly expressed in neurons, existing in an equilibrium of soluble cytosolic and membrane-bound α-helical forms^8^. However, recombinant αSyn monomers easily undergo aggregation in physiological buffer conditions and form β-sheet-rich amyloid structures that resemble those observed in LB in diseased brains^9,10^. These αSyn aggregates generated de novo from recombinant αSyn under controlled conditions (commonly referred to as preformed fibrils; PFF) have been widely used to study different aspects of LBD over the past decade. PFF works as a template to recruit and seed the misfolding of endogenous αSyn in cultured cells and neurons^11,12^. Similarly, PFF inoculation induces αSyn inclusions in animal brains and is accompanied subsequently by intercellular transmission of the αSyn pathology^13,14^, a phenomenon also inferred from autopsy studies on subjects with PD^15–17^.

Previous studies have demonstrated the presence of distinct αSyn conformers, or strains, among αSyn aggregates generated in vitro as well as those extracted from synucleinopathy brains, that have different biological activities^18–26^. Cryogenic electron microscopy (Cryo-EM) studies have also revealed atomic-level structural differences among human αSyn preformed fibrils (hPFF), LBD αSyn fibrils, and MSA αSyn fibrils^27,28^. Based on these observations, it has been hypothesized that the clinical and pathological heterogeneity in synucleinopathies arises from distinct αSyn strains.

Despite the clear influence of fibril structure on αSyn in vitro and in vivo pathological activity with respect to seeding potency, inclusion morphology, cell tropism, and biophysical signatures, attempts to generate recombinant fibrils that faithfully recapitulate the properties of brain-derived αSyn aggregates have been met with limited success. Furthermore, even though brain-derived αSyn aggregates can template the fibrillization of recombinant αSyn, as observed in seeded aggregation assays or protein misfolding cyclic amplification-based strategies using brain lysates or cerebrospinal fluid, fibrils generated in this manner remain conformationally and functionally distinct from brain-derived αSyn aggregates^27–31^.

Highlighting these differences, we recently demonstrated differences in pathological activity in vitro and conformational features between hPFF and αSyn aggregates extracted from LBD brains (LB-αSyn)^32^. In order to bridge this gap and to overcome the limited availability and low seeding activity of LB-αSyn compared to αSyn aggregates extracted from MSA brains^20^, we established a method for amplifying LB-αSyn using recombinant human αSyn monomers. By optimizing the stoichiometry of LB-αSyn and recombinant αSyn monomers in assembly mixtures, this template-seeded amplification approach successfully replicated the conformational features of original LB-αSyn and unique pathological activity in vitro^32^.

Here, we aimed to uncover whether or not the distinct pathological features induced by the LB-αSyn strain are preserved in the brain. Our findings demonstrate conspicuous differences in pathological features induced by hPFF vs. ampLB in the brains of wild-type (WT) mice. We then developed a novel LBD mouse model through the injection of amplified LB (ampLB) into mice expressing only human αSyn (Thy1:*SNCA*/*Snca*^–/–^ mice). The comparisons of the ampLB-injected Thy1:*SNCA*/*Snca*^–/–^ mice with LBD subjects, hPFF-injected Thy1:*SNCA*/*Snca*^–/–^ mice, and old Thy1:*SNCA*/*Snca*^–/–^ mice with spontaneous αSyn pathology further solidify the role of conformation as a determinant of αSyn pathological activity. Our results provide a novel approach for improving currently available LBD models induced by PFF injection or αSyn overexpression.

## Results

### Lewy body amplification increases αSyn pathology induced in WT mice

**Fig. 1:**
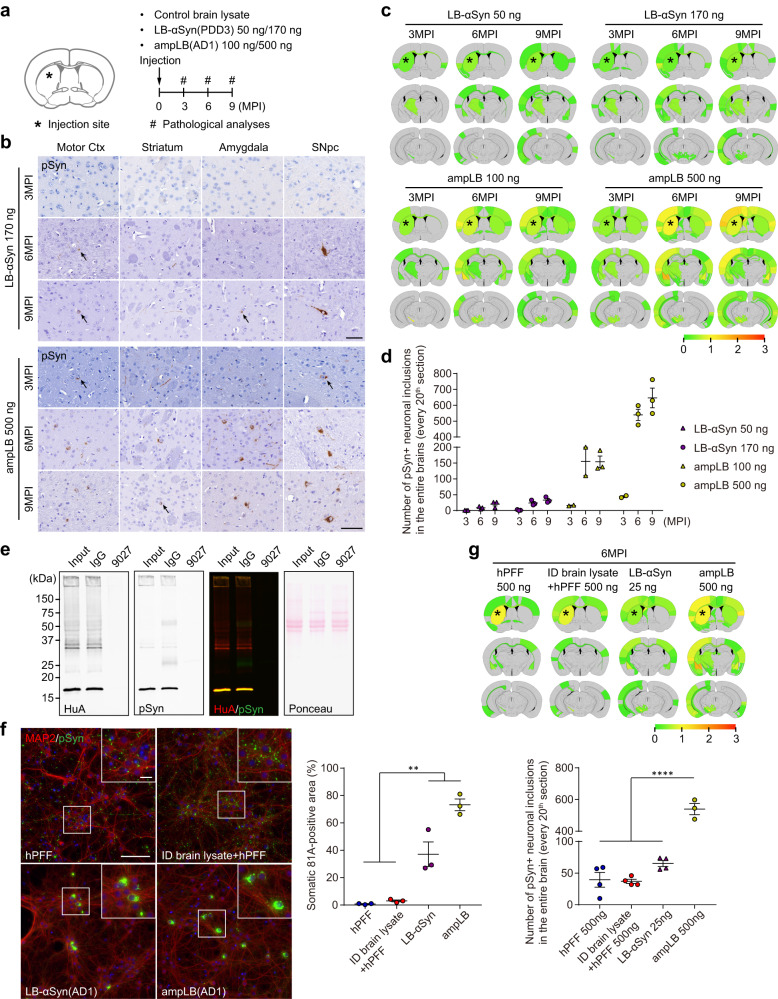
Lewy body amplification increases αSyn pathology induced in WT mice. **a** Schematic representation of experimental design. **b** Immunohistochemistry with an anti-pSyn antibody (EP1536Y). Arrows indicate pSyn-positive neuronal somatic inclusions. Motor Ctx, Motor cortex; SNpc, substantia nigra pars compacta. Scale bar 50 µm. **c** Heat-map colors represent the extent of pSyn-positive pathology. **d** Number of pSyn-positive neuronal somatic inclusions in the entire brains (LB-αSyn 50 ng and 170 ng, *n* = 3; ampLB 100 ng, *n* = 2, 2, 3; ampLB 500 ng, *n* = 2, 3, 3 biologically independent samples). **e** Western blot analysis of AD1 brain lysate immunodepleted with a control mouse IgG or an anti-human αSyn antibody 9027. Immunoblots with anti-human αSyn (HuA) and pSyn (81 A) antibodies. Images are representative of two independent experiments. **f** Left panels: Mouse primary hippocampal neurons treated with hPFF, immunodepleted (ID) AD1 brain lysate mixed with hPFF, LB-αSyn (AD1), and ampLB (AD1). Note that the ID brain lysate mixed with hPFF and LB-αSyn contains almost the same contaminants. Scale bars 100 µm, 20 µm (inset). Immunocytochemistry with anti-MAP2 and pSyn (81 A) antibodies. Right panel: Percent of total pSyn-positive pathology in neuronal somatic inclusions (*n* = 3 independent wells per group). One-way ANOVA with a Tukey’s post hoc test was performed (hPFF vs. LB-αSyn, ampLB *p* = 0.0038, <0.0001; ID brain lysate + hPFF vs. LB-αSyn, ampLB *p* = 0.0057, <0.0001). **g** Upper panel: Heat-map colors represent the extent of pSyn-positive pathology at 6MPI. Lower panel: Number of pSyn-positive neuronal somatic inclusions in the entire brains (hPFF 500 ng, ID brain lysate + hPFF 500 ng, and LB-αSyn 25 ng, *n* = 4; ampLB 500 ng, *n* = 3 biologically independent samples). One-way ANOVA with a Tukey’s post hoc test was performed (hPFF 500 ng, ID brain lysate + hPFF 500 ng, LB-αSyn 25 ng vs. ampLB 500 ng *p* < 0.0001). Data are represented as mean ± SEM. Source data are provided as a Source Data file.

We previously observed that striatal injection of LB-αSyn only induced sparse αSyn pathology when injected into WT mouse brains^20^. We therefore sought to augment the level of αSyn pathology by increasing the amount of LB-αSyn injected and by injecting ampLB (Fig. 1a). Our LB-αSyn amplification methodology increases the αSyn concentration 20 times, up to 200 ng/µl^32^. We injected LB-αSyn at two doses: 50 ng, the same dose as we injected previously^20^ or 170 ng, the maximum dose achievable with brain-derived lysate (Supplementary Table 2). For ampLB injection, we injected either 100 ng, a comparable quantity to LB-αSyn injection, or 500 ng, the maximum dose we could inject with ampLB. We analyzed αSyn pathology at 3, 6, and 9 months post-injection (MPI) and quantified the number of phospho-serine129 αSyn (pSyn)-positive neuronal somatic inclusions. Both LB-αSyn and ampLB-induced αSyn pathology in a dose- and time-dependent manner, though the higher concentration (170 ng) of LB-αSyn only induced mild αSyn pathology throughout the brain (Fig. 1b–d). Meanwhile, ampLB induced more αSyn pathology than LB-αSyn, and 500 ng of ampLB induced the most severe αSyn pathology (Fig. 1b–d). Based on these results, we decided to inject 500 ng of ampLB in mice for most experiments using WT mice. We did not observe any αSyn pathology in control brain lysate-injected samples at any time points.

LB-αSyn only comprises less than 2% of the total protein in LBD brain lysates (Supplementary Table 2). To examine the effects of contaminants on αSyn pathogenesis in mouse primary neurons and mouse brains, we immunodepleted αSyn in LBD brain lysate (Fig. 1e). hPFF and immunodepleted brain lysate mixed with hPFF-induced neurite-dominant αSyn pathology, while LB-αSyn and ampLB-induced soma-dominant αSyn pathology in mouse primary neurons (Fig. 1f). For mouse brain injection, we used the following materials: hPFF, immunodepleted brain lysate mixed with hPFF (designated as mixed material), original LB-αSyn used to generate ampLB, and ampLB. hPFF- and mixed material-injected WT mice showed similar distribution and amount of αSyn pathology, while ampLB-injected samples induced significantly higher αSyn pathology than them at 6MPI (Fig. 1g). These results suggest that contaminants contained in LB-αSyn have minimal effects on hPFF-induced αSyn pathology both in vitro and in vivo. Moreover, the results also suggest distinct pathological activity between hPFFs and ampLBs in the presence of similar contaminants, further supporting the faithful amplification of LB-αSyn.

### Distinct biological activity between hPFF and ampLB in WT mice

**Fig. 2:**
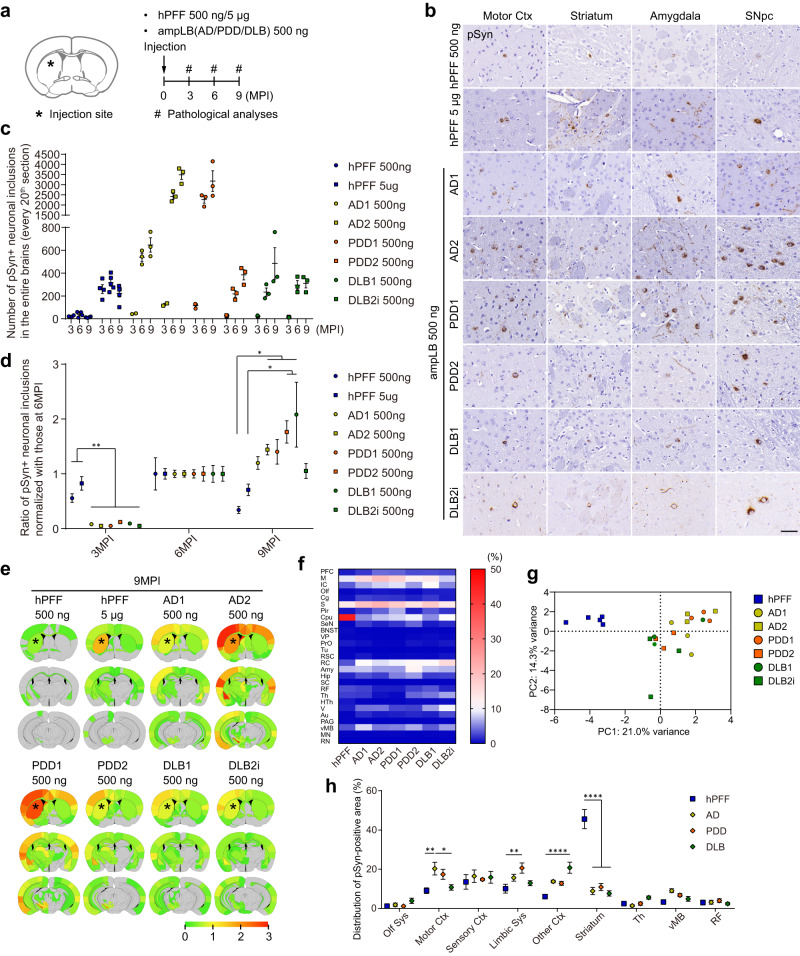
Differences in seeding activity and distribution of αSyn pathology between hPFF and ampLB in WT mice. **a** Schematic representation of experimental design. **b** Immunohistochemistry with an anti-pSyn antibody (EP1536Y) at 6MPI. Motor Ctx, motor cortex; SNpc, substantia nigra pars compacta. Scale bar 50 µm. **c**, **d** Number of pSyn-positive neuronal somatic inclusions in the entire brain (hPFF 500 ng, *n* = 4; hPFF 5 µg, *n* = 4, 5, 5; AD1, AD2 500 ng, *n* = 2, 3, 3; PDD1, PDD2, DLB1, DLB2i 500 ng, *n* = 3 biologically independent samples). **d** Ratio of numbers of pSyn-positive neuronal somatic inclusions normalized with those at 6MPI (3MPI, *n* = 4, 4, 2, 2, 3, 3, 3, 3; 6MPI, *n* = 4, 5, 3, 3, 3, 3, 3, 3; 9MPI, *n* = 4, 5, 3, 3, 3, 3, 3, 3 biologically independent samples). One-way ANOVA with a Tukey’s post hoc test was performed (3MPI: hPFF 500 ng vs. AD1, AD2, PDD1, PDD2, DLB1, DLB2i 500 ng *p* = 0.0093, 0.0055, 0.0016, 0.0068, 0.0038, 0.0016; hPFF 5 µg vs. AD1, AD2, PDD1, PDD2, DLB1, DLB2i 500 ng, *p* < 0.0001; 9MPI: hPFF 500 ng vs. AD2, PDD1, PDD2, DLB1 500 ng *p* = 0.0371, 0.0478, 0.0041, 0.0005; hPFF 5 µg vs. PDD2, DLB1 500 ng *p* = 0.0362, 0.0038). **e** Heat-map colors represent the extent of pSyn-positive pathology at 9MPI. **f** Heat-map colors represent the proportion of pSyn-positive area in each brain region shown in Supplementary Fig. 5b at 9MPI. **g** Primary component analysis of the distribution of pSyn-positive pathology at 9MPI. **h** Distribution of pSyn-positive pathology in the brain systems at 9MPI (hPFF, *n* = 5; AD, PDD, DLB, *n* = 6 biologically independent samples). Olf Sys, olfactory system; Motor Ctx, motor cortex; Sensory Ctx, sensory cortex; Limbic Sys, limbic system; Th, thalamus; vMB, ventral midbrain; RF, reticular formation. Two-way ANOVA with Sidak’s post hoc test was performed (interaction *p* < 0.0001, Motor Ctx: hPFF vs. AD *p* = 0.0017; AD vs. DLB *p* = 0.0103; Limbic Sys: hPFF vs. PDD *p* = 0.0054; Other Ctx: hPFF vs. DLB *p* < 0.0001; Striatum: hPFF vs. AD, PDD, DLB *p* < 0.0001). Data are represented as mean ± SEM. Source data are provided as a Source Data file.

To further characterize the pathological activity of ampLB in WT mice, we generated brain lysates from 2 AD cases, 2 PDD cases, and 2 DLB cases, and determined possible differences among ampLB generated from different LBD cases. We validated ampLB from each brain lysate by testing its ability to induce distinct morphology of αSyn pathology in mouse primary neurons compared to multiple hPFF batches (Supplementary Fig. 1a, c). We then injected WT mice with ampLB preparations (500 ng) or hPFF (500 ng and 5 µg), and analyzed them histologically at 3, 6, and 9MPI (Fig. 2a). hPFF-injected samples showed the highest amount of αSyn pathology in brains at 6MPI. In contrast, all the ampLB-injected samples showed little αSyn pathology at 3MPI but showed much more pathology at 6MPI, which was further increased at 9MPI (Fig. 2b–d). Based on the number of neuronal somatic inclusions at 6MPI, ampLB preparations showed 5–50 times more seeding activity than hPFF. Compared with the numbers of neuronal inclusions at 6MPI, hPFF, and ampLB preparations induced 60–80% and 5–10% of the inclusions at 3MPI, respectively, suggesting that ampLB takes longer to induce αSyn pathology than hPFF (Fig. 2d). The numbers of neuronal inclusions induced by ampLB-injection in the SNpc also jumped at 6MPI but were decreased at 9MPI (Supplementary Fig. 2a, b). The numbers of tyrosine hydroxylase (TH)-positive neurons in the ipsilateral SNpc were significantly decreased compared with those in the contralateral SNpc in AD2 and PDD1 ampLB-injected samples at 9MPI (Supplementary Fig. 2c), suggesting that the decrease in numbers of neuronal inclusions was caused by TH-positive neuron loss. Biochemical analysis revealed that injected ampLB, which were generated from human αSyn monomers, were almost completely degraded by 3MPI (Supplementary Fig. 3). Meanwhile, mouse αSyn and pSyn, including their monomeric and oligomeric forms, were increased over time, suggesting that pSyn-positive pathology induced by ampLB-injection was composed of endogenous mouse αSyn.

Since we observed ~10-fold differences in seeding activity in WT mouse brains among ampLB preparations, we sought to identify the main contributing factors. One preparation each of ampLB generated from AD and PDD brain lysate (AD2 and PDD1) showed high seeding activity, suggesting that the differences in seeding activity were not disease-specific. There was no correlation between seeding activity and age at onset or disease duration (Supplementary Tables 1 and 2). One factor we found was the total protein in ampLB preparations, which was negatively correlated with the seeding activity both in vivo and in vitro, albeit not reaching statistical significance (Supplementary Fig. 4a–c). However, the in vivo and in vitro seeding activity were positively correlated (Supplementary Fig. 4d). An independent set of ampLB preparations also showed some variability in in vitro seeding activity and a trend towards an inverse correlation between the seeding activity and total protein levels (Supplementary Fig. 4e, f).

We next analyzed the distribution of αSyn pathology in hPFF- and ampLB-injected samples at 6 and 9MPI. hPFF-injected samples showed the most severe pathology in the striatum, the injection site, while all the ampLB-injected samples showed the most severe pathology in some cortical areas and the amygdala (Fig. 2e and Supplementary Fig. 5a). We quantitatively measured proportion of pSyn-positive area based on the classification of brain regions shown in Supplementary Fig. 5b. Heatmap and principal component analysis showed the differences in distribution of αSyn pathology between hPFF- and ampLB-injected samples (Fig. 2f, g; Supplementary Fig. 5c, d). We further classified brain regions into several brain systems to statistically analyze the differences in the distribution of αSyn pathology induced by hPFF and LBD (including AD, PDD, and DLB) ampLB preparations. The significant differences observed in some brain systems further validated the difference in the distribution of αSyn pathology between hPFF- and ampLB-injected samples (Fig. 2h; Supplementary Fig. 5e). Importantly, we did not observe clear differences among LBD (AD, PDD, and DLB) from these analyses.

**Fig. 3:**
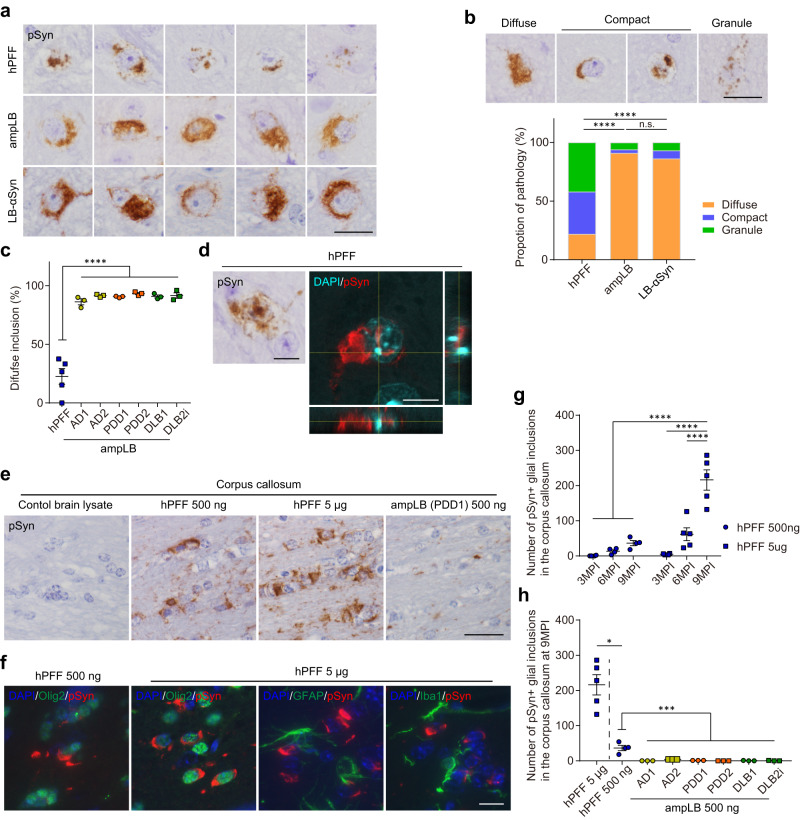
Differences in morphology of neuronal inclusions and cell-type preference between hPFF and ampLB in WT mice. **a** High-magnification image of pSyn-positive neuronal inclusions in the SN of hPFF-, ampLB-, and LB-αSyn-injected mice at 6MPI. Images are representative of 9, 18, and 7 biologically independent samples, respectively. Scale bar 20 µm. **b** Upper panels: Representative images of diffuse, compact, and granular pSyn-positive inclusions. Lower panels: Comparison of the proportions of diffuse, compact, and granular inclusions (hPFF, *n* = 50 from 9 biologically independent samples; ampLB, *n* = 959 from 18 biologically independent samples; LB-αSyn, *n* = 29 from 7 biologically independent samples). Neuronal inclusions with visible nuclei were evaluated. A two-sided Fisher’s exact test was performed between two groups for differences in the percentages of diffuse and non-diffuse inclusions (hPFF vs. ampLB, LB-αSyn *p* < 0.0001). Scale bar 20 µm. **c** Percent of diffuse inclusions in hPFF- and ampLB-injected mice (hPFF *n* = 5; AD1, AD2, PDD1, PDD2, DLB1, DLB2i, *n* = 3 biologically independent samples). One-way ANOVA with a Tukey’s post hoc test was performed (hPFF vs. AD1, AD2, PDD1, PDD2, DLB1, DLB2i *p* < 0.0001). **d** Immunohistochemistry and *z*-stack confocal microscopy images showing pSyn-positive intranuclear inclusions in hPFF-injected mice. Images are representative of two independent experiments. Scale bars 10 µm. **e** Immunohistochemistry with a pSyn antibody (EP1536Y) in the corpus callosum at 9MPI. Scale bar 20 µm. **f** Double immunofluorescence for Olig2 (green) and pSyn (81 A, red), GFAP (green) and pSyn (EP1536Y, red), and Iba1 (green) and pSyn (81 A, red). Scale bar 10 µm. **g** Number of pSyn-positive oligodendroglial inclusions in the corpus callosum (hPFF 500 ng, *n* = 4; hPFF 5 µg, *n* = 4, 5, 5 biologically independent samples). Two-way ANOVA with Sidak’s post hoc test was performed (interaction *p* = 0.0001, hPFF 500 ng 3, 6, 9MPI, hPFF 5 µg 3, 6MPI vs. hPFF 5 µg 9MPI *p* < 0.0001). **h** Number of pSyn-positive oligodendroglial inclusions in the corpus callosum at 9MPI (hPFF 5 µg, *n* = 5; hPFF 500 ng, *n* = 4; AD1, AD2, PDD1, PDD2, DLB1, DLB2i, *n* = 3 biologically independent samples). A two-tailed Mann–Whitney test was performed (hPFF 5 µg vs. hPFF 500 ng *p* = 0.0159). One-way ANOVA with a Tukey’s post hoc test was performed (hPFF 500 ng vs. AD1, PDD1, PDD2, DLB1, DLB2i *p* < 0.0001; hPFF 500 ng vs. AD2 *p* = 0.0002). Data are represented as mean ± SEM. Source data are provided as a Source Data file.

Next, we examined the morphology of pSyn-positive neuronal inclusions induced by hPFF, ampLB, and LB-αSyn injection. hPFF-injected samples showed various morphology of neuronal inclusions, while ampLB- and LB-αSyn-injected samples mostly showed diffuse somatic pathology (Fig. 3a). We classified neuronal inclusions into three types based on their morphology: diffuse somatic pathology (diffuse), isolated compact pathology (compact), and granular pathology (granule) (Fig. 3b). The proportion of each morphology seen in hPFF-injected samples was clearly different from those seen in ampLB- and LB-αSyn-injected samples. All the ampLB-injected samples showed mostly diffuse pathology, and the proportion of this pathology was significantly different from that of hPFF-injected samples (Fig. 3c). Aside from morphological differences, we found that ~10% of neurons with pSyn-positive somatic inclusions also contained intranuclear inclusions in hPFF-injected mice (6/50 and 23/164 in the SNpc and motor cortex, respectively, at 6MPI), while these were rarely observed in ampLB-injected ones (Fig. 3d).

Additionally, we found cell-type preference as another difference between hPFF- and ampLB-induced pathology. hPFF induced glial inclusions in WT mouse brains, especially in the corpus callosum, in a dose- and time-dependent manner, the observation we previously reported for mouse αSyn PFF^33^ (Fig. 3e, g). We further confirmed that these glial inclusions were present in Olig2-positive oligodendroglia, but not in glial fibrillary acidic protein (GFAP)-positive astrocytes or ionized calcium-binding adaptor protein-1 (Iba1)-positive microglia (Fig. 3f). Significantly, glial inclusions were rarely observed in all the ampLB-injected samples up to 9MPI (Fig. 3h).

### Modeling LBD in Thy1:*SNCA*/*Snca*^–/–^ mice

**Fig. 4:**
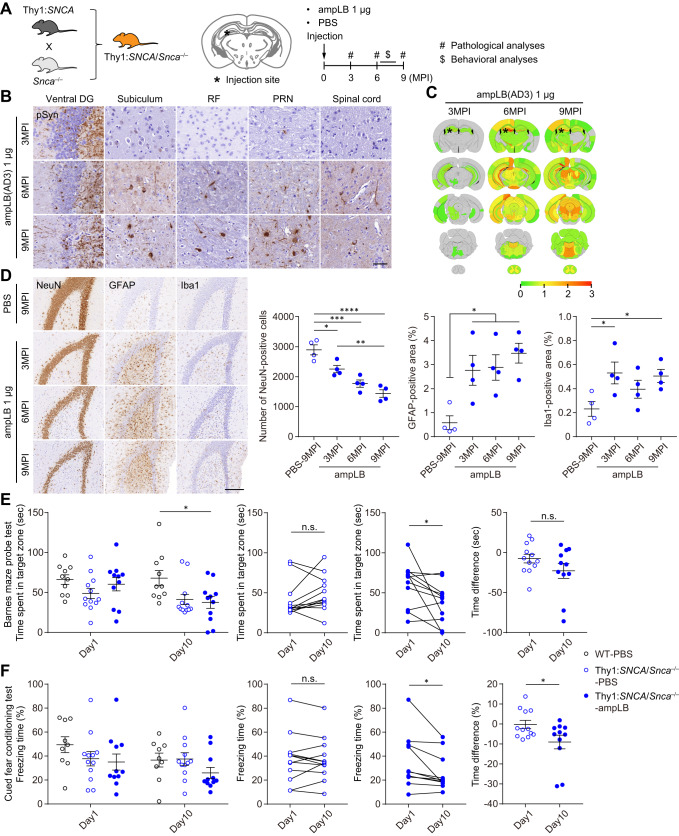
Modeling LBD in Thy1:*SNCA*/*Snca*^–/–^ mice. **A** Schematic representation of experimental design. **B** Immunohistochemistry with an anti-pSyn antibody (EP1536Y) on the ipsilateral side. Images for 3, 6, and 9MPI are representative of 2, 3, and 2 biologically independent samples, respectively. Ventral DG, ventral dentate gyrus; RF, reticular formation; PRN, pontine reticular formation. Scale bar 50 µm. **C** Heat-map colors represent the extent of pSyn-positive pathology. **D** Left panels: Immunohistochemistry with anti-NeuN, GFAP, and Iba1 antibodies in the ipsilateral ventral dentate gyrus. Scale bar 200 µm. Right panels: Number of NeuN-positive cells, GFAP-positive area, and Iba1-positive area in the ipsilateral ventral dentate gyrus (*n* = 4 biologically independent samples per group). One-way ANOVA with a Tukey’s post hoc test was performed (Number of NeuN-positive cells: PBS-9MPI vs. ampLB-3, 6, 9MPI *p* = 0.0249, 0.0004, <0.0001; ampLB-3MPI vs. ampLB-9MPI *p* = 0.0052; GFAP-positive area: PBS-9MPI vs. ampLB-3, 6, 9MPI *p* = 0.0324, 0.0237, 0.0051). One-way ANOVA with a Dunnett’s post hoc test was performed (PBS-9MPI vs. ampLB-3, 9MPI *p* = 0.0303, 0.0476). **E** Barnes maze probe test at 7MPI (WT-PBS, *n* = 10; Thy1:*SNCA*/*Snca*^–/–^-PBS, *n* = 12; Thy1:*SNCA*/*Snca*^–/–^-ampLB, *n* = 11 biologically independent samples). Left panel: Time spent in a target zone on day 1 and day 10. One-way ANOVA with a Tukey’s post hoc test was performed for day 10 (WT-PBS vs. Thy1:*SNCA*/*Snca*^–/–^-ampLB *p* = 0.0299). Middle panels: Time spent in a target zone between day 1 and day 10. A two-tailed paired *t*-test was performed (Thy1:*SNCA*/*Snca*^–/–^-ampLB *p* = 0.0342). Right panel: Difference in time spent in a target zone between day 1 and day 10. A two-tailed unpaired Student’s *t*-test was performed (*p* = 0.161). **F** Cued fear conditioning test at 8MPI (WT-PBS, *n* = 9; Thy1:*SNCA*/*Snca*^–/–^-PBS, *n* = 12; Thy1:*SNCA*/*Snca*^–/–^-ampLB, *n* = 11 biologically independent samples). Left panel: Freezing time during auditory cue at day and day. Middle panels: freezing time between day 1 and day 10. A two-tailed paired *t*-test was performed (Thy1:*SNCA*/*Snca*^–/–^-ampLB *p* = 0.0249). Right panel: Difference in freezing time between day 1 and day 10. A two-tailed unpaired Student’s *t*-test was performed (*p* = 0.0356). Data are represented as mean ± SEM. Source data are provided as a Source Data file.

Because of the significant differences in biological activity between hPFF and the LB-αSyn strains in cultured cells and WT mouse brains, we sought to generate a novel LBD animal model that would recapitulate the spread of the LB-αSyn strain in the brain. Considering possible differences in pathology induced by human vs. mouse αSyn, we generated mice that only express human αSyn by crossing mice expressing human αSyn under a mouse Thy1 promoter (Thy1:*SNCA* mice) with *Snca* knock-out (*Snca*^–/–^) mice (Fig. 4A). As the transgene of Thy1:*SNCA* mice is inserted in the X chromosome, we used only male Thy1:*SNCA*/*Snca*^–/–^ mice for this study. They expressed high levels of αSyn compared with WT mice, especially in the brainstem, cerebellum, and spinal cord, but low levels of αSyn in the SN (Supplementary Fig. 6a). Western blot analysis showed Thy1:*SNCA*/*Snca*^–/–^ mice expressed ~ 4-fold more αSyn than WT mice in the entire brains (Supplementary Fig. 6b). We injected 1 µg of ampLB into the dorsal hippocampus of Thy1:*SNCA*/*Snca*^–/–^ mice and conducted pathological analyses at 3, 6, and 9MPI (Fig. 4A; Supplementary Fig. 1b, c). These mice showed severe αSyn pathology in the ipsilateral ventral dentate gyrus (DG) but only showed little pathology in other brain regions at 3MPI (Fig. 4B, C). They showed very severe pathology in the ipsilateral hippocampus and severe pathology in some brain regions at 6MPI, with more pathology, especially in the brainstem at 9MPI. Although Thy1:*SNCA*/*Snca*^–/–^ mouse neurons showed stronger pSyn immunoreactivity than WT mouse neurons, ampLB-induced αSyn inclusions were clearly distinguished based on their even stronger pSyn staining intensity and morphology. We evaluated pathological changes in the ipsilateral ventral DG because of its remarkable αSyn pathology during the time course (Fig. 4D). The number of NeuN-positive neurons in ampLB-injected animals was significantly decreased compared with that of PBS-injected animals from 3MPI, and that was further decreased in a time-dependent manner. Both GFAP- and Iba1-positive areas were significantly increased compared with PBS-injected samples from 3MPI, suggesting reactive astrogliosis and microglial activation.

We conducted behavioral analyses on ampLB-injected Thy1:*SNCA*/*Snca*^–/–^ mice together with PBS-injected WT mice and Thy1:*SNCA*/*Snca*^–/–^ mice between 6 and 8MPI. The open field test showed longer total distance traveled in Thy1:*SNCA*/*Snca*^–/–^ mice than that of PBS-injected WT mice, indicative of their hyperactivity (Supplementary Fig. 6c). The Y-maze test showed no significant differences in alternation among the groups (Supplementary Fig. 6d). In the probe trial of the Barnes maze, time spent in target zone was not different between day1 and day10 in PBS-injected Thy1:*SNCA*/*Snca*^–/–^ mice, while that was significantly decreased in ampLB-injected Thy1:*SNCA*/*Snca*^–/–^ mice at day10 compared with day1 (Fig. 4E; Supplementary Fig. 6e). Likewise, in the cued fear conditioning test, freezing time during auditory cue was not different between day1 and day10 in PBS-injected Thy1:*SNCA*/*Snca*^–/–^ mice, while that was significantly decreased in ampLB-injected Thy1:*SNCA*/*Snca*^–/–^ mice at day10 compared with day1 (Fig. 4F; Supplementary Fig. 6f). The time difference between day1 and day10 was significantly decreased in ampLB-injected Thy1:*SNCA*/*Snca*^–/–^ mice compared with PBS-injected Thy1:*SNCA*/*Snca*^–/–^ mice. These results suggest that injection of ampLB into the hippocampus induced impairment of spatial and cued fear memory retention.

### Pathological and phenotypic features of hPFF-injected Thy1:*SNCA*/*Snca*^–/–^ mice and old Thy1:*SNCA*/*Snca*^–/–^ mice with spontaneous αSyn pathology

**Fig. 5:**
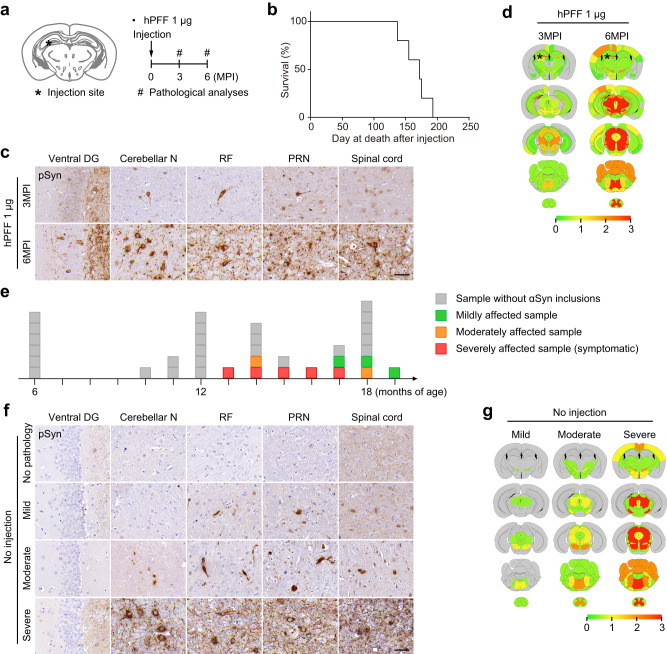
hPFF injection into Thy1:*SNCA*/*Snca*^–/–^ mice induces pathological and phenotypic features distinct from ampLB. **a** Schematic representation of experimental design. **b** Survival curve of hPFF-injected Thy1:*SNCA*/*Snca*^–/–^ mice (*n* = 5 biologically independent samples). **c** Immunohistochemistry with an anti-pSyn antibody EP1536Y on the ipsilateral side. Ventral DG, ventral dentate gyrus; Cerebellar N, cerebellar nuclei; RF, reticular formation; PRN, pontine reticular nucleus. Scale bar 50 µm. **d** Heat-map colors represent the extent of pSyn-positive pathology. **e** Schematic representation of age and severity of αSyn pathology. Each box represents one Thy1:*SNCA*/*Snca*^–/–^ mouse (*n* = 35 biologically independent samples). The color indicates the severity of αSyn pathology (gray, sample without αSyn inclusions; green, mildly affected sample; orange, moderately affected sample; red, severely affected sample). **f** Immunohistochemistry with an anti-pSyn antibody EP1536Y on the ipsilateral side. Arrows indicate pSyn-positive inclusions. Scale bar 50 µm. **g** Heat-map colors represent the extent of pSyn-positive pathology.

Aside from ampLB-injection, we also applied 1 µg hPFF-injection to Thy1:*SNCA*/*Snca*^–/–^ mice (Fig. 5a). hPFF-injected Thy1:*SNCA*/*Snca*^–/–^ mice showed rapid spread of αSyn pathology in the brain with very severe pathology in the ipsilateral DG and severe pathology in the brainstem regions at 3MPI, followed by very severe pathology in the ipsilateral DG, brainstem, and spinal cord at 6MPI (Fig. 5c, d). Mice showed paralysis and ataxia from ~5MPI and did not survive beyond 6MPI (Fig. 5b).

We found that some of the old Thy1:*SNCA*/*Snca*^–/–^ mice without ampLB- or hPFF-injection developed spontaneous αSyn pathology over 13 months of age. We first observed that some Thy1:*SNCA*/*Snca*^–/–^ mice exhibited paralysis and ataxia and then died (Fig. 5e). Their motor dysfunction resembled that of hPFF-injected mice. All of the mice showed very severe pathology, especially in the brainstem and spinal cord (Fig. 5f, g). We also sacrificed old asymptomatic Thy1:*SNCA*/*Snca*^–/–^ mice and found less severe pathology in some of them. We classified them into mildly and moderately affected cases based on the severity of αSyn pathology (Fig. 5e–g).

### Similarities and differences in pathological features among Thy1:*SNCA*/*Snca*^–/–^ mouse models and LBD subjects

**Fig. 6:**
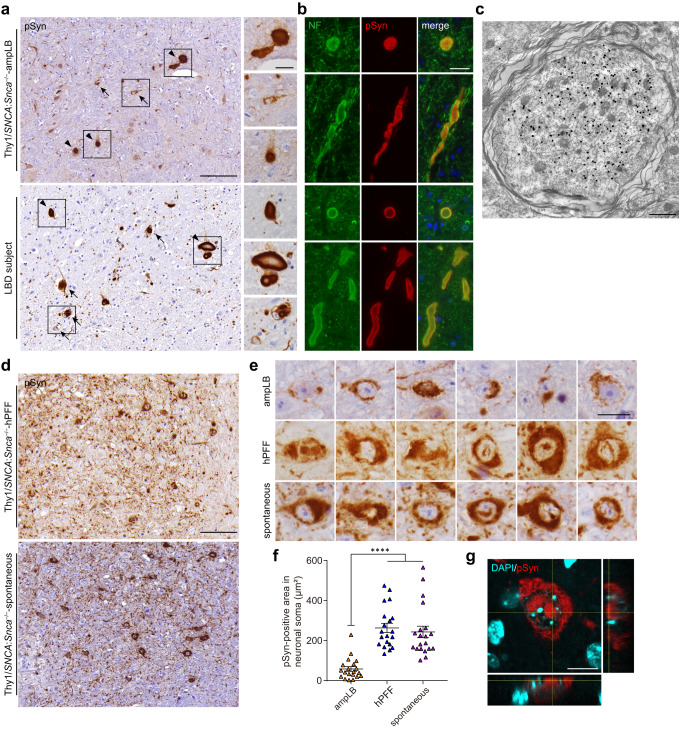
Similarities and differences in pathological characteristics among Thy1:*SNCA*/*Snca*^–/–^ mouse models and LBD subjects. **a** Immunohistochemistry with an anti-pSyn antibody (EP1536Y) in the pons of an ampLB-injected Thy1:*SNCA*/*Snca*^–/–^ mouse (Thy1:*SNCA*/*Snca*^–/–^-ampLB) at 9MPI and a LBD subject. Images of Thy1:*SNCA*/*Snca*^–/–^-ampLB are representative of 4 biologically independent samples. Images of other LBD subjects are shown in Supplementary Fig. 7. Arrows and arrowheads indicate pSyn-positive neuronal inclusions and axonal swelling-like structures, respectively. Scale bars 100 µm, 20 µm (inset). **b** Double immunofluorescence for neurofilament (NF, green) and pSyn (#64, red). Upper panels: an ampLB-injected Thy1:*SNCA*/*Snca*^–/–^ mouse, Lower panels: a LBD subject. Images are representative of two independent experiments. Scale bar 20 µm. **c** Immunoelectron micrograph of an axonal swelling in the pons of an ampLB-injected Thy1:*SNCA*/*Snca*^–/–^ mouse with immunogold-labeled pSyn (EP1536Y). Images are representative of two independent experiments. Scale bar 1 µm. **d** Immunohistochemistry with an anti-pSyn antibody EP1536Y in the pons of a hPFF-injected Thy1:*SNCA*/*Snca*^–/–^ mouse (Thy1:*SNCA*/*Snca*^–/–^-hPFF) at 6MPI and a Thy1:*SNCA*/*Snca*^–/–^ mouse with spontaneous αSyn pathology (Thy1:*SNCA*/*Snca*^–/–^-spontaneous) at 13 months of age. Images of both Thy1:*SNCA*/*Snca*^–/–^-hPFF and Thy1:*SNCA*/*Snca*^–/–^-spontaneous are representative of 4 biologically independent samples. Scale bar 100 µm. **e** High-magnification images of pSyn-positive neuronal inclusions in the pons of ampLB-injected Thy1:*SNCA*/*Snca*^–/–^ mice at 6MPI, hPFF-injected Thy1:*SNCA*/*Snca*^–/–^ mice at 6MPI, and Thy1:*SNCA*/*Snca*^–/–^ mice with spontaneous αSyn pathology. Scale bar 20 µm. **f** PSyn-positive area in neuronal soma (*n* = 20 from 2 biologically independent samples per group). One-way ANOVA with a Tukey’s post hoc test was performed (ampLB vs. hPFF, spontaneous *p* < 0.0001). **g** *z*-stack confocal microscopy images showing pSyn-positive intranuclear inclusions in hPFF-injected mice. Scale bar 10 µm. Data are represented as mean ± SEM. Source data are provided as a Source Data file.

We then evaluated the similarities and differences in pathological features among Thy1:*SNCA*/*Snca*^–/–^ mouse models and LBD subjects. We first examined the pons, where all the Thy1:*SNCA*/*Snca*^–/–^ mouse models and most LBD subjects show abundant αSyn pathology. AmpLB-injected Thy1:*SNCA*/*Snca*^–/–^ mice and LBD subjects, including those used for brain extractions, showed large spheroid-like structures, which were sometimes larger in diameter than small neurons (Fig. 6a; Supplementary Fig. 7a). Consistent with this finding, these objects were also observed in other brain regions (Supplementary Fig. 7b, c). These spheroid-like structures were positive for neurofilament, suggesting axonal swellings in addition to cell body inclusions (Fig. 6b). Consistent with these observations, pSyn-positive expanded myelinated axons filled with filamentous structures were observed in ampLB-injected Thy1:*SNCA*/*Snca*^–/–^ mice by immunoelectron microscopy (Fig. 6c). Meanwhile, hPFF-injected Thy1:*SNCA*/*Snca*^–/–^ mice and old Thy1:*SNCA*/*Snca*^–/–^ mice with spontaneous αSyn pathology showed neuronal somatic αSyn inclusions with abundant fine neuritic αSyn pathology (Fig. 6d; Supplementary Fig. 7b).

hPFF-injected Thy1:*SNCA*/*Snca*^–/–^ mice and Thy1:*SNCA*/*Snca*^–/–^ mice with spontaneous αSyn pathology showed dense neuronal pSyn-positive inclusions, which were larger than those of ampLB-injected Thy1:*SNCA*/*Snca*^–/–^ mice (Fig. 6e, f). Further, ~25% of the neuronal inclusions contained intranuclear inclusions (34/128 and 30/125 in the pons of hPFF-injected Thy1:*SNCA*/*Snca*^–/–^ mice and Thy1:*SNCA*/*Snca*^–/–^ mice with spontaneous αSyn pathology, respectively. Fig. 6e, g). We also examined αSyn inclusions in neuronal soma in ampLB- and hPFF-injected Thy1:*SNCA*/*Snca*^–/–^ mice by immunoelectron microscopy. Bundles of pSyn-positive filamentous structures were mostly observed in the cytosol in both ampLB- and hPFF-injected Thy1:*SNCA*/*Snca*^–/–^ mice (Supplementary Fig. 8a–c).

### AmpLB-induced pathological αSyn in Thy1:*SNCA*/*Snca*^–/–^ mice maintains the biological and conformational features of the original LB-αSyn

**Fig. 7:**
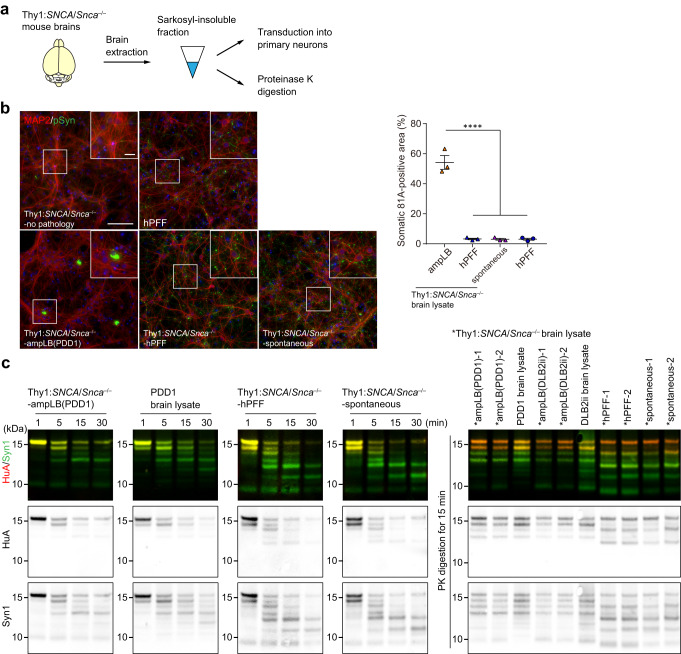
AmpLB-induced pathological αSyn in Thy1:*SNCA*/*Snca*^–/–^ mice maintains the biological and conformational features of original LB-αSyn. **a** Schematic representation of experimental design. **b** Left panels: Mouse primary hippocampal neurons treated with brain lysates of Thy1:*SNCA*/*Snca*^–/–^ mice without αSyn pathology (Thy1:*SNCA*/*Snca*^–/–^-no pathology), ampLB-injected Thy1:*SNCA*/*Snca*^–/–^ mice (Thy1:*SNCA*/*Snca*^–/–^-ampLB), hPFF-injected Thy1:*SNCA*/*Snca*^–/–^ mice (Thy1:*SNCA*/*Snca*^–/–^-hPFF), and old Thy1:*SNCA*/*Snca*^–/–^ mice with spontaneous αSyn pathology (Thy1:*SNCA*/*Snca*^–/–^-spontaneous), and hPFF. Immunocytochemistry with MAP2 and pSyn (81 A) antibodies. Scale bars 100 µm, 20 µm (inset). Right panel: Percent of total pSyn-positive pathology in somatic inclusions (*n* = 3 independent wells per group). One-way ANOVA with a Tukey’s post hoc test was performed (Thy1:*SNCA*/*Snca*^–/–^-ampLB vs. Thy1:*SNCA*/*Snca*^–/–^-hPFF, Thy1:*SNCA*/*Snca*^–/–^-spontaneous, hPFF *p* < 0.0001). Scale bar 10 µm. **c** Proteinase K (PK) digestion on Thy1:*SNCA*/*Snca*^–/–^ mouse brain lysate and LBD brain lysate. Left panels: Samples were subjected to PK digestion for 1, 5, 15, and 30 min, followed by western blot analysis with anti-human αSyn antibodies HuA and Syn1. Right panel: Samples were subjected to PK digestion for 15 min, followed by western blot analysis with HuA and Syn1 antibodies. Images are representative of two independent experiments. Data are represented as mean ± SEM. Source data are provided as a Source Data file.

Finally, we performed biochemical extraction on Thy1:*SNCA*/*Snca*^–/–^ mouse brains to examine the biological and conformational features of αSyn aggregates (Fig. 7a; Supplementary Fig. 9a). As expected, little sarkosyl-insoluble αSyn was obtained from the brains of Thy1:*SNCA*/*Snca*^–/–^ mice without spontaneous αSyn pathology (Supplementary Fig. 9b and Supplementary Table 3). Meanwhile, a considerable amount of sarkosyl-insoluble, pSyn-positive αSyn aggregates was obtained from the brains of ampLB- and hPFF-injected Thy1:*SNCA*/*Snca*^–/–^ mice and Thy1:*SNCA*/*Snca*^–/–^ mice with spontaneous αSyn pathology (Supplementary Fig. 9b and Supplementary Table 3). We first transduced those αSyn aggregates into mouse primary neurons to see their biological activity. αSyn aggregates from Thy1:*SNCA*/*Snca*^–/–^ mice without spontaneous pathology induced little pSyn-positive pathology. However, those from ampLB-injected Thy1:*SNCA*/*Snca*^–/–^ mice induced soma-dominant αSyn pathology, while those from hPFF-injected Thy1:*SNCA*/*Snca*^–/–^ mice and Thy1:*SNCA*/*Snca*^–/–^ mice with spontaneous αSyn pathology induced neurite-dominant αSyn pathology like hPFF (Fig. 7b).

In order to determine whether the conformational properties of ampLB continued to be propagated in vivo, we next conducted partial proteinase K (PK) digestion on αSyn aggregates isolated from Thy1:*SNCA*/*Snca*^–/–^ mouse brains and the original LB-αSyn used for the ampLB-injection. Digestion reactions were stopped at 1, 5, 15, and 30 min. αSyn aggregates from ampLB-injected Thy1:*SNCA*/*Snca*^–/–^ mice and the original LB-αSyn showed similar digestion profiles, which were different from those from hPFF-injected Thy1:*SNCA*/*Snca*^–/–^ mice and Thy1:*SNCA*/*Snca*^–/–^ mice with spontaneous αSyn pathology (Fig. 7c). We conducted PK digestion on multiple samples from each group for 15 min and ran them in a single gel. The samples from each group showed similar digestion profiles and largely reflected the treatment group from which they were derived. Altogether, these results suggest that pathological αSyn aggregates in ampLB-injected Thy1:*SNCA*/*Snca*^–/–^ mice maintained the biological and conformational features of the original LB-αSyn. Moreover, these features are clearly different from those in hPFF-injected Thy1:*SNCA*/*Snca*^–/–^ mice and Thy1:*SNCA*/*Snca*^–/–^ mice with spontaneous αSyn pathology. Interestingly, the pathological αSyn aggregates in hPFF-injected Thy1:*SNCA*/*Snca*^–/–^ mice and Thy1:*SNCA*/*Snca*^–/–^ mice with spontaneous αSyn pathology showed similar biological and conformational features to each other.

## Discussion

Although multiple previous studies have investigated the effect of injecting LBD brain lysates into animal brains, a unique pathological profile induced by LB-αSyn has not been reported thus far^14,20,21,26,34–36^. This may be partly due to the low αSyn yield and relatively low seeding activity of LB-αSyn compared with αSyn extracted from MSA brains. In this study, we described the detailed biological activity of a prototypic LB-αSyn strain in animal brains by using an amplification methodology that we recently established^32^.

This approach allowed us to compare the pathological features induced by the same doses of hPFF and ampLB. We report here differences in biological activity between hPFF and ampLB in WT mouse brains that are reflected in seeding activity, latency in inducing pathology, distribution of pathology, morphology of neuronal inclusions, and cell-type preference. Meanwhile, we did not observe obvious disease-specific differences in biological activity among ampLB preparations generated from AD, PDD, and DLB brain lysates. These results are consistent with a recent Cryo-EM study showing essentially undistinguishable atomic-level core structures among PD, PDD, and DLB αSyn filaments^28^, and suggest that the differences in clinical and pathological features among these LBD may arise from other factors than strain differences alone. However, we observed up to a ~ 10-fold difference in seeding activity among ampLB preparations. In other words, ampLB preparations generated from a single brain lysate and those generated from different brain lysates of a single case can show considerable variability. High seeding activity is important to sufficiently induce αSyn pathology and model LBD in animals. In this study, we found a negative correlation between seeding activity and total protein (likely reflecting contaminants) in ampLB preparations, but further studies are needed to precisely identify the factors affecting seeding activity. Nonetheless, our results showed that seeding activity in vitro may help predict the amount of pathology induced in animal brains.

Currently, PFF-injected animals are widely used for modeling and understanding LBD. However, significant differences in biological activity between hPFF and the LB-αSyn strain prompted us to generate a novel LBD model employing ampLB as a pathological seed in a host that only expresses human αSyn. AmpLB-injected Thy1:*SNCA*/*Snca*^–/–^ mice recapitulated several characteristics of LBD including the presence of pSyn-positive neuronal inclusions, neuron loss, glial activation, and behavioral abnormalities. As a comparison, we also challenged Thy1:*SNCA*/*Snca*^–/–^ mice with hPFF-injection, which induced rapid spread of αSyn pathology. Interestingly, we found that a subset of old Thy1:*SNCA*/*Snca*^–/–^ mice developed αSyn inclusions without ampLB- or hPFF-injection, which was not reported in the original Thy1:*SNCA* line^37^. The exacerbation of αSyn pathology in Thy1:*SNCA*/*Snca*^–/–^ mice compared with Thy1:*SNCA* mice may arise from at least two potential possibilities: (1) differences in genetic background and (2) the absence of endogenous mouse αSyn expression. The latter is consistent with previous studies showing that deletion of endogenous mouse αSyn accelerates aggregation of human αSyn overexpressed in cultured cells and in mice^38,39^.

We examined the similarities and differences in brain pathological features and biological and conformational features of αSyn aggregates in brain lysates among the Thy1:*SNCA*/*Snca*^–/–^ mouse models and LBD subjects. AmpLB-injected Thy1:*SNCA*/*Snca*^–/–^ mice and LBD subjects showed similarities to each other, while they showed clear differences from hPFF-injected Thy1:*SNCA*/*Snca*^–/–^ mice and old Thy1:*SNCA*/*Snca*^–/–^ mice with spontaneous αSyn pathology. These results have potentially significant implications. First, αSyn aggregates in ampLB-injected Thy1:*SNCA*/*Snca*^–/–^ mouse brains propagate the biological and conformational features of original LB-αSyn throughout the process of amplification, brain injection, and in vivo incubation. This further suggests that the similarities of pathological features between ampLB-injected Thy1:*SNCA*/*Snca*^–/–^ mice and LBD subjects come from their strain similarities. However, we did not observe αSyn inclusions in ampLB-injected Thy1:*SNCA*/*Snca*^–/–^ mice that morphologically resemble the brainstem LB consisting of a central core and surrounding halo in LBD subjects. Longer incubation times may be needed for such prototypical LB to be developed, or this may be attributed to different pathological responses between mice and humans. The other important implication is that αSyn aggregates in Thy1:*SNCA*/*Snca*^–/–^ mice with spontaneous αSyn pathology showed different features from LB-αSyn even though both Thy1:*SNCA*/*Snca*^–/–^ mice and LBD subjects spontaneously develop human αSyn aggregates. These results suggest that the LB-αSyn strain is not replicated by human αSyn overexpression in mice. In other words, the introduction of the LB-αSyn strain into human αSyn-expressing mice is required to faithfully recapitulate LBD pathology. Further studies are needed to investigate what factors are required to generate the LB-αSyn strain confirmation in animals and cultured cells expressing human αSyn.

This study includes several limitations. First, we were unable to evaluate the size distribution of αSyn aggregates in brain lysates and ampLB after sonication by electron microscopy because of the presence of contaminants. Second, conformations of αSyn aggregates were indirectly determined by partial PK digestion, but not examined at atomic resolution by Cryo-EM. Third, further expansion of ampLB through additional rounds of amplification was not demonstrated. These limitations are being addressed in ongoing studies.

In conclusion, we detail the similarities and differences in features of αSyn brain pathology and pathological αSyn aggregates among mouse models and LBD subjects, highlighting the ampLB-injection as a novel strategy for improvement upon conventional PFF-injection or αSyn overexpression animal models. Considering the unique pathological mechanisms induced by the LB-αSyn strain, ampLB-injected animal models will provide new opportunities to identify therapeutic targets, develop diagnostic imaging tools, and test disease-modifying therapies for LBD.

## Methods

### Ethics statement

All animal procedures were approved by the University of Pennsylvania Institutional Animal Care and Use Committee and conformed to the National Institute of Health Guide for Care and Use of Laboratory Animals. The use of postmortem tissue in this study was approved by the Institutional Review Boards of the University of Pennsylvania. Written informed consent for autopsy and analysis of tissue sample data was obtained either from patients themselves or their next of kin.

### Mice

Female C57BL/6 C3H (B6C3) mice at 2 months of age purchased from Charles River were used for the experiments in Figs. 1–3. Thy1:*SNCA*/*Snca*^–/–^ mice were generated in the Center for Neurodegenerative Disease Research (CNDR) by crossing Thy1:*SNCA* mouse line 61^37^ on a DBA background with *Snca*^–/–^ mice^40^ on a B6C3 background. Because the transgene is inserted in the X chromosome, only male Thy1:*SNCA*/*Snca*^–/–^ mice were used for this study. To generate male Thy1:*SNCA*/*Snca*^–/–^ mice, female Thy1:*SNCA*/*Snca*^–/–^ mice were crossed with male *Snca*^–/–^ mice. Male Thy1:*SNCA*/*Snca*^–/–^ mice and male B6C3 mice as WT mice at 2–3 months of age were used for the experiments in Figs. 4–7. Mice were housed at a controlled temperature (26–34 °C) and relative humidity (30–70%) under a 12-h light/dark cycle with free access to food and water.

### Primary hippocampal neuron cultures

Primary mouse neurons were prepared from the hippocampus of embryonic day E16–E18 CD1 mouse embryos as described previously^12^. Dissociated hippocampal neurons were plated at 100,000 cells/well (24-well plate) or 17,500 cells/well (96-well plate) in neuron media (Neurobasal medium, Thermo Fisher #21103049) supplemented with B27 (Thermo Fisher #17504044), 2 mM GlutaMax (Thermo Fisher #35050061), and 100 U/ml penicillin/streptomycin (Thermo Fisher #15140122).

### Human patient samples

Detailed clinical characteristics (disease duration, age at death, site of onset, etc.) were ascertained from an integrated neurodegenerative disease database in CNDR at the University of Pennsylvania. Frozen and paraffinized postmortem brain samples were obtained from patient brain donors who underwent autopsy at CNDR between 2002 and 2018. More details on these patients are found in Supplementary Table 1.

### Biochemical extraction of sarkosyl-insoluble αSyn from human and mouse brains

Biochemical brain extraction was conducted as described previously with minor modifications^32^. All human brain tissues were obtained from the CNDR brain bank^41^. Fontal cortex tissues with a high burden of αSyn pathology from patients with AD, PDD, and DLB were identified by postmortem neuropathological examination^5^. Biochemical extraction of human brains was performed as described previously^32^. In brief, 5–10 g of frontal cortical gray matter was homogenized in five volumes (w/v) of 1% (v/v) Triton X-100-containing high-salt (HS) buffer (50 mM Tris–HCl pH 7.4, 750 mM NaCl, 10 mM NaF, 5 mM ethylenediaminetetraacetic acid [EDTA]) with protease and protein phosphatase inhibitors, incubated on ice for 20 min, and centrifuged at 180,000 × *g* for 30 min. The pellets were then re-extracted with five volumes of 1% (v/v) Triton X-100-containing HS buffer, followed by sequential extraction with five volumes of HS buffer with 30% (w/v) sucrose for myelin floatation. The pellets were then re-suspended and homogenized in 2% (w/v) sarkosyl-containing HS buffer, rotated at room temperature for 1 h or at 4 °C overnight, and centrifuged at 180,000 × *g* for 30 min. The resulting sarkosyl-insoluble pellets were washed once with Dulbecco’s PBS (DPBS, Corning #21-031-CV) and re-suspended in DPBS by sonication (QSonica Microson XL-2000; 60 pulses, setting 2, 0.5 s per pulse). This suspension termed the “sarkosyl-insoluble fraction” or “brain lysate” contained pathological αSyn referred to as “LB-αSyn” and was used for the experiments. Mouse brain extraction was performed with the same protocol for human brain extraction except that only one round of extraction with 1% Triton X-100-containing HS buffer was performed (Supplementary Fig. 9a). The Triton X-100-soluble fraction was used for the experiments in Supplementary Fig. 6b. The concentrations of αSyn in the sarkosyl-insoluble fractions were determined by sandwich enzyme-linked immuno sorbent assay (ELISA) (see ‘Sandwich ELISA’), and the protein concentrations were examined by bicinchoninic acid (BCA) assay (Supplementary Tables  2 and 3).

### Sandwich ELISA

Sandwich ELISA was conducted as described previously^32^. To measure the concentration of αSyn in brain lysates, 384-well Nunc Maxisorp clear plates were coated with 100 ng (30 μl per well) of an anti-human αSyn antibody Syn9027 (CNDR) in sodium carbonate buffer, pH 9.6, and incubated overnight at 4 °C. The plates were washed 4 times with PBS containing 1% (v/v) Tween 20 (PBS-T), and blocked using Block Ace solution (AbD Serotec) overnight at 4 °C. Brain lysates were sonicated with a Diagenode Biorupter sonicator (20 min, 30 s on, 30 s off, 10 °C, high setting), serially diluted in PBS and added to each well. The plates were incubated overnight at 4 °C. The recombinant human αSyn monomer and hPFF were used as standards. The plates were then washed with PBS-T and an anti-human αSyn antibody MJFR1 (Abcam #ab138501, 1:1000) or an anti-human αSyn antibody HuA (CNDR, 1:2000) was added to each well and incubated at 4 °C overnight. After washing, a secondary antibody conjugated with horse radish peroxidase (Cell Signaling Technology #7074, 1:10000) was added to the plates followed by incubation for 1 h at 37 °C. Following another wash, the plates were developed for 10–15 min using 1-Step Ultra TMB-ELISA substrate solution (Thermo Fisher Scientific #37574, 30 μl per well), the reaction was quenched using 10% phosphoric acid, and plates were read at 450 nm on a Molecular Devices Spectramax M5 plate reader.

### Recombinant αSyn purification and in vitro PFF generation

Purification of recombinant human αSyn and generation of hPFF was conducted as described previously^42^. The pRK172 plasmid containing a full-length human αSyn gene was transformed into BL21 (DE3) RIL-competent E. coli (Agilent Technologies #230245). A single colony from the transformed bacteria was expanded in Terrific Broth (12 g/l of Bacto-tryptone, 24 g/l of yeast extract 4% (v/v) glycerol, 17 mM KH_2_PO_4_ and 72 mM K_2_HPO_4_) with ampicillin. Bacterial pellets from the growth were sonicated, and the sample was boiled to precipitate undesired proteins. The supernatant was dialyzed with 10 mM Tris, pH 7.6, 50 mM NaCl, 1 mM EDTA overnight. Protein was filtered with a 0.22 μm filter and concentrated using Amicon Ultra-15 centrifugal filters (Millipore Sigma #UFC901008). Protein was then loaded onto a Superdex 200 column and 1 ml fractions were collected. Fractions were run on sodium dodecyl sulfate-polyacrylamide gel electrophoresis (SDS-PAGE) and stained with Coomassie blue to select fractions that were highly enriched in αSyn. These fractions were combined and dialyzed in 10 mM Tris, pH 7.6, 50 mM NaCl, 1 mM EDTA overnight. Dialyzed fractions were applied to a HiTrap Q HP anion-exchange column (GE Healthcare #17115301) and run using a linear gradient from 25 mM NaCl to 1 M NaCl. Collected fractions were run on SDS-PAGE and stained with Coomassie blue. Fractions that were highly enriched in αSyn were collected and dialyzed with DPBS. Protein was filtered through a 0.22 μm filter and concentrated to 5 mg/ml (αSyn) with Amicon Ultra-15 centrifugal filters. αSyn monomer was aliquoted and frozen at −80 °C. For preparation of PFF, αSyn monomer was shaken at 1000 rpm for 7 days. Conversion to PFF was validated by sedimentation at 100,000 × *g* for 60 min and by Thioflavin S staining.

### In vitro amplification of LB-αSyn from brain lysates

LB-αSyn amplification was performed as described previously with minor modifications^32^. LB-αSyn from brain lysates of patients pathologically confirmed with AD, PDD, or DLB was sonicated with a Diagenode Biorupter sonicator (20 min, 30 s on, 30 s off, 10 °C, high setting). The amplification reaction was set up with 5% LB-αSyn (calculated based on sandwich ELISA), 95% human αSyn monomer, and DPBS (pH 7.4, without Mg^2+^ and Ca^2+^) at the final αSyn concentration of 200 or 400 ng/µl. The solution was incubated at 37 °C with constant agitation at 1000 rpm for 14 days. The resulting material (ampLB) was transduced into mouse primary hippocampal neurons to verify the success of amplification of LB-αSyn.

### αSyn transduction into mouse primary neurons

αSyn transduction into mouse primary hippocampal neurons was performed as described previously^32^. hPFF, LB-αSyn, ampLB, and αSyn aggregates extracted from Thy1:*SNCA*/*Snca*^–/–^ mouse brains were diluted in DPBS and sonicated with a Diagenode Biorupter sonicator (20 min, 30 s on, 30 s off, 10 °C, high setting). Neurons were then treated with a noted dose of the αSyn preparations at 7 days in vitro (DIV), fixed, and immunostained at 14 days post-treatment (21 DIV). The amounts of αSyn transduced in Fig. 1f, Supplementary Fig. 1, and in Supplementary Fig. 4e were 25 and 50 ng/well, respectively. For the treatment with αSyn aggregates extracted from Thy1:*SNCA*/*Snca*^–/–^ mouse brains in Fig. 7b, the brain lysates containing 25 ng of αSyn or up to 6.4 μg of total protein per well were transduced to avoid significant toxicity of contaminants for cultured neurons^32^. The resulting amounts of αSyn were 2.4 ng/well (Thy1:*SNCA*/*Snca*^–/–^ mice without αSyn pathology), 19 ng/well (ampLB-injected Thy1:*SNCA*/*Snca*^–/–^ mice), and 25 ng/well (hPFF-injected Thy1:*SNCA*/*Snca*^–/–^ mice and Thy1:*SNCA*/*Snca*^–/–^ mice with spontaneous αSyn pathology).

### Immunocytochemistry and quantification of neuron pathology

Immunocytochemistry and quantification of pSyn-positive neuronal pathology were performed as described previously^32^. Mouse primary neurons cultured in 96 wells were washed with PBS once and fixed at DIV 21 with 4% (w/v) PFA, 4% (w/v) sucrose, and 1% (v/v) Triton X-100 in PBS. After PBS washes, cells were blocked with 3% (w/v) bovine serum albumin (BSA), 5% (w/v) fetal bovine serum in DPBS for 1 h at room temperature, then incubated with an anti-pSyn antibody 81 A (CNDR, 1:5000) and an anti-microtubule associated protein 2 (MAP2) antibody #17028 (CNDR, 1:3000) at 4 °C overnight. The cells were washed 5 times with PBS and incubated with secondary antibodies conjugated with Alexa fluor 488 or 594 (Invitrogen #A11029, #11037, 1:1000) for 2 h at room temperature. After washing with PBS, the cells were incubated in DAPI solution (Thermo Fisher #D21490, 1:10,000 in PBS) after staining with secondary antibodies, and the plates were sealed with adhesive covers. The 96-well plates were scanned with an In Cell Analyzer 2200 (GE Healthcare) with a 10 × or 40 × objective and analyzed using the accompanying software (In Cell Toolbox Analyzer). Quantitation of total 81 A signal and the amount of somatic 81 A signal was calculated using Cell Profiler ver. 3.1.9 (The Broad Institute). The fraction of somatic inclusions was calculated as the 81 A signal intensity (density times area) of somatic objects divided by the total 81 A signal intensity. Data are reported as the average of 3 replicate wells for each treatment sample.

### Western blotting

Protein concentrations of samples were determined with a BCA assay kit (Fisher #23223 and 23224) using BSA as a standard (Thermo Fisher #23210). Samples were normalized for total protein content and boiled with SDS-sample buffer for 10 min. Samples (10 μg total protein) were separated on 12.5% SDS-polyacrylamide gels and transferred onto 0.2 μm nitrocellulose membranes. For the samples digested by PK, NuPAGE Novex 12% Bis–Tris gels (Invitrogen) were used. The membranes were fixed with 4% (w/v) paraformaldehyde in tris-buffered saline (TBS) for 30 min to prevent detachment of αSyn from the blotted membranes. Ponceau staining was performed to visualize the protein transferred to the membranes. After being blocked in 5% (w/v) non-fat milk in TBS for 30 min, the membranes were probed at 4 °C overnight with the following primary antibodies: an anti-human αSyn antibody HuA (CNDR, 1:500–3000), an anti-pSyn antibody 81 A (CNDR, 1:5000), an anti-human αSyn-specific antibody LB509 (CNDR, 1:100), an anti-mouse αSyn-specific antibody D37A6 (Cell signaling #4179, 1:1000), an anti-αSyn antibody Syn1 (BD transduction #610787, 1:500–1000), and an anti-pSyn antibody EP1536Y (abcam #ab51253, 1:5000). Primary antibodies were detected using IRDye 800 (Li-Cor #925-32210) and IRDye 680 (Li-Cor #925-68071) labeled secondary antibodies, scanned on a Li-Cor Odyssey Imaging System and analyzed using Image Studio software (Li-Cor Biosciences). Densitometric analyses were performed using ImageJ (NIH).

### Immunodepletion of αSyn from brain lysate

An anti-human αSyn monoclonal antibody 9027 was covalently conjugated to Dynabeads M-280, tosylactivated (Invitrogen #14204) per the manufacturer’s instructions. Immunodepletion of αSyn was performed by incubating diluted AD1 brain lysate (10 ng/μl of αSyn, 50 μl total dose) with anti-αSyn antibody–bead complexes containing 68 μg of the antibody at 37 °C for 1 h with constant rotation. The immunodepleted fraction was separated from the antibody–bead complex using a magnet. Mock immunodepletion was performed using an equal amount of a control mouse IgG antibody (Jackson Immuno Research). The brain lysate was immunodepleted with the anti-αSyn antibody and the control antibody was used for western blot analysis. The αSyn-depleted brain lysate (2.5 μl) mixed with hPFFs (500 ng) was used for mouse brain injection. Diluted AD1 brain lysate (10 ng/μl of αSyn, 2.5 μl total dose) and amplified LB-αSyn generated from AD1 brain lysate (200 ng/μl of αSyn, 2.5 μl total dose) were used for injection as comparisons. Those three injection materials contained almost the same contaminants.

### Partial PK digestion of αSyn aggregates in brain lysate

Sarkosyl-insoluble fractions from LBD brains and Thy1:*SNCA*/*Snca*^–/–^ mouse brains were prepared by biochemical brain extraction. For partial PK digestion, 50 ng of αSyn from each sample was sonicated with a Diagenode Biorupter sonicator (20 min, 30 s on, 30 s off, 10 °C, high setting) and mixed with 0.2 μg of PK in DPBS to a final volume of 50 μl and incubated at 37 °C for 1, 5, 15, and 30 min. The reaction was stopped with 1 mM PMSF. The samples were boiled with SDS-sample buffer for 10 min and resolved on NuPAGE Novex 12% Bis–Tris gels (Invitrogen). Transferred nitrocellulose membranes were probed with an anti-human αSyn antibody HuA (CNDR, 1:500) and an anti-αSyn antibody Syn1 (BD transduction #610787, 1:500).

### Stereotaxic inoculation of mouse brains

Stereotaxic surgery was performed as described previously with minor modifications^13^. Mice anesthetized with ketamine–xylazine–acepromazine underwent stereotaxic injection. A 30-gauge syringe was used for brain lysate injection, and a 33-gauge syringe was used for hPFF- and ampLB-injection. For pathological analysis in Figs. 1–3, WT mice received a unilateral injection of 2.5 μl of hPFF (200 ng/μl or 2 µg/μl of αSyn, 500 ng or 5 µg total dose) or ampLB (200 ng/μl of αSyn, 500 ng total dose) into the dorsal striatum (coordinates: 0.2 mm relative to bregma; 2.0 mm from midline; –3.2 mm beneath the skull surface). For biochemical analysis in Supplementary Fig. 3, WT mice received a bilateral injection of 2.5 μl of ampLB (400 ng/μl of αSyn, 1 µg total dose) into the dorsal striatum. For pathological, behavioral, and biochemical analysis in Figs. 4–7, Thy1:*SNCA*/*Snca*^–/–^ and WT mice received a unilateral injection of 2.5 μl of hPFF (400 ng/μl of αSyn, 1 µg total dose), ampLB (400 ng/μl of αSyn, 1 µg total dose), or PBS into the dorsal hippocampus (coordinates: –2.5 mm relative to bregma; 2.0 mm from midline; –2.4 mm beneath the skull surface). The mice were sacrificed at indicated time points and were subjected to histological and biochemical analyses.

### Immunohistochemical analysis of injected mouse brains

Mice were deeply anesthetized with ketamine–xylazine–acepromazine. Following intracardial perfusion with PBS, mice were perfused with 15 ml of fixative containing 4% (w/v) PFA in PBS. The brains were collected and immersed in 4% (w/v) PFA in PBS at 4 °C overnight. The brains were embedded in paraffin and then sectioned with a thickness of 6 μm. For immunohistochemical analyses, the sections were incubated at 4 °C for 2 days with following primary antibodies: an anti-pSyn antibody EP1536Y (Abcam #ab51253, 1:20,000), an anti-TH antibody (Sigma-Aldrich #T2928, 1:10,000), an anti-NeuN antibody (Sigma-Aldrich #MAB377, 1:2000), an anti-GFAP antibody 2.2B10 (CNDR, 1:5000), and an anti-Iba1 antibody (Wako #019-19741, 1:2000). Biotinylated secondary antibodies (Vector laboratories #BA-2000, #BA-1000, #BA4000, 1:1000) were used for generating diaminobenzidine reaction product, and nuclei were counterstained with hematoxylin.

To quantify the amount of αSyn pathology, every 20th paraffin section throughout the brains was stained with an anti-pSyn antibody EP1536Y, and pSyn-positive neuronal somatic inclusions with visible nuclei were manually counted. To assess the distribution and severity of αSyn pathology, semi-quantitative analyses were performed for pSyn-positive pathology on the five coronal sections (2.80, 0.26, −1.58, −2.92, and −4.04 mm relative to bregma), and color-coded onto heat maps (Figs. 1c, g, 2e, 4c, 5d, g; Supplementary Fig. 5a). The extent of αSyn pathology was graded as 0–3 (0, no pathology; 0.5, mild; 1, moderate; 2, severe; 3, very severe) based on the criteria described previously^43^, and averaged across samples for each brain region. To quantitatively assess the distribution of pSyn-positive area, each brain region shown in Supplementary Fig. 5b was measured using QuPath software^44^. The proportion of pSyn-positive area was averaged across samples for each brain region, and color-coded onto heat maps (Fig. 2f; Supplementary Fig. 5c). Primary component analysis of the distribution of pSyn-positive pathology was performed using GraphPad Prism Software, Version 9.

To assess dopaminergic neuron loss in the SNpc, every 20th section was stained with an anti-TH antibody throughout the SNpc. The numbers of TH-positive cells with visible nuclei were manually counted. To assess neuron loss and glial activation in the ventral DG, sections at −3.52 mm relative to bregma were stained with anti-NeuN, GFAP, and Iba1 antibodies. The numbers of NeuN-positive neurons were automatically counted and the GFAP- and Iba1-positive area was measured using QuPath software. Sections were examined with a BX43 microscope (Olympus) or a Panoramic 250 (3DHISTECH) scanner.

For immunofluorescence, the sections were incubated at 4 °C for 2 days with the following primary antibodies: an anti-pSyn antibody EP1536Y (Abcam #ab51253, 1:5000), an anti-pSyn antibody 81 A (CNDR, 1:2000), an anti-pSyn antibody #64 (Wako #015-25191, 1:2000), anti-TH antibody (Sigma-Aldrich #T2928, 1:2000), an anti-Olig2 antibody (Millipore #AB9610, 1:500), an anti-GFAP antibody 2.2B10 (CNDR, 1:2000), an anti-Iba1 antibody (Wako #019-19741, 1:1000), and an anti-phosphorylated neurofilament antibody TA51 (CNDR, 1:500). Fluorescent dye-conjugated secondary antibodies (Invitrogen #A11029, #A11034, #A11006, #A11032, #A11037, 1:1000) were used, and nuclei were stained with DAPI. Sections were examined with an Eclipse Ni microscope (Nikon) or a TCS SP8 WLL Confocal with STED 3X (Leica).

### Immunoelectron microscopy

Preparative procedures for pre-embedding immunoelectron microscopy have been described in detail elsewhere^45^. Mice were deeply anesthetized with ketamine–xylazine–acepromazine. Following intracardial perfusion with 0.1 M phosphate buffer (PB) (pH 7.2), mice were perfused with 30 ml of fixative containing 4% (w/v) PFA and 0.05% (w/v) glutaraldehyde in 0.1 M PB. The brains were removed from the skull immediately after perfusion, post-fixed in 4% (w/v) PFA overnight at 4 °C, and coronally sectioned at 50 μm by a vibratome (VT1200S; Leica). Sections were pre-incubated for 30 min in PBS containing 20% (v/v) normal donkey serum (Jackson ImmunoResearch Laboratories), 0.3% (v/v) Photo-flo 600 (Kodak), incubated overnight at 4 °C with 13 μg/ml anti-pSyn rabbit antibody EP1536Y (Abcam #ab51253) in PBS containing 2% (v/v) normal donkey serum and 0.3% (v/v) Photo-flo 600, and then washed twice with PBS. Subsequently, after incubation overnight at 4 °C with 1/100-diluted gold-conjugated anti-rabbit IgG goat antibody (Ultra-small Gold Reagent) in PBS containing 2% (v/v) normal donkey serum and 0.3% (v/v) Photo-flo 600, the sections were post-fixed with 1% (w/v) glutaraldehyde in 0.1 M PB for 10 min. After washing with distilled water, gold particles were developed with a silver enhancement kit (R-GENT SE-EM). The sections were then washed with 0.1 M PB, placed for 40 min in 0.1 M PB containing 1% (w/v) osmium tetroxide, counterstained for 30 min with 1% (w/v) uranyl acetate, dehydrated, and flat-embedded in epoxy resin (Luveak 812; Nacalai Tesque). After polymerization of the resin, approximately 70-nm-thick ultrathin sections were cut with an ultramicrotome (UC6; Leica), stained briefly with 1% (w/v) uranyl acetate and 1% (w/v) lead citrate, and observed with an electron microscope (HT7700; Hitachi).

### Behavioral analysis

WT mice injected with PBS, Thy1:*SNCA*/*Snca*^–/–^ mice injected with PBS, and Thy1:*SNCA*/*Snca*^–/–^ mice injected with ampLB were subjected to behavioral tests from 6 to 8MPI. Before every test, the mice were habituated to the experimental environment for more than 30 min. Samples that encountered technical problems were removed from the analyses.

#### Open field

Mice were placed at the center of the field inside an open field apparatus (36 × 36 cm) and allowed to move freely for 15 min. The distance traveled and time spent in the center area (18 × 18 cm) were recorded using video tracking software EthoVision XT 15 (Noldus).

#### Y-maze

Mice were placed at the end of one arm of the Y-maze apparatus (San Diego Instruments) and allowed to move freely for 5 min. The distance traveled and series of arm entries were recorded using video tracking software EthoVision XT 15 (Noldus). An alternation was defined as entries into all three arms on consecutive occasions. The number of maximum alternations was therefore the total number of arm entries minus two, and the percentage of alternations was calculated.

#### Barnes maze

The Barnes maze test was conducted on a white circular surface with 20 holes equally spaced along the perimeter (Supplementary Fig. 6e). For acquisition trials, a shelter was placed under one of the holes, i.e., the “target hole”. Mice were placed in the center and allowed to move freely for up to 3 min. The latency to reach the target, the number of holes visited other than the target, and the distance traveled were recorded using the video tracking software EthoVision XT 15 (Noldus). Mice were subjected to acquisition trials twice a day for 7 days, and then to probe tests 24 h and 10 days after the last acquisition trial. In the probe tests without the shelter, mice were placed in the center and allowed to move freely for 3 min. The time spent around each hole, the number of visiting each hole, and the distance traveled were recorded. “Target zone” was defined as the target hole plus the 2 holes on either side of the target hole.

#### Contextual fear conditioning

Conditioning and test sessions were performed in a standard operant chamber (Med Associates) equipped with a tone generator and house light. Mice were handled for habituation in front of the apparatus for 2 min per day for 3 days. On day 0, conditioning was performed (Supplementary Fig. 6f). Mice were placed in a test chamber inside a sound-attenuated cabinet and allowed to explore freely for 150 s. A white noise, which is a conditioned stimulus, was presented for 30 s, followed by a foot shock (2 s, 1 mA) serving as an unconditioned stimulus. On days 1 and 10, contextual fear memory and auditory-cued fear memory tests were performed. For the contextual fear memory test, mice were placed in the same chamber in the same context as the conditioning and immobile time and distance traveled were recorded for 5 min. For the auditory-cued fear memory test, mice were placed in a different chamber in a different context from the conditioning. Mice were allowed to move freely for 2.5 min, and then the white noise was presented for 2.5 min. Immobile time and distance traveled were recorded automatically.

### Quantification and statistical analyses

A number of samples or animals analyzed in each experiment, statistical analysis performed, as well as *p-*values for all results, are described in the figure legends. For all the in vivo and in vitro experiments, “n” represents the number of animals and wells, respectively. An *F*-test, a Brown–Forsythe test, or Bartlett’s test was performed to evaluate the differences in variances. An unpaired, two-tailed Student’s *t*-test, a two-tailed paired *t*-test, or a Mann–Whitney test was used to determine statistical significance between the two groups. One- or two-way analysis of variance (ANOVA) with Dunnett’s, Tukey’s, or Sidak’s multiple comparison tests were used to determine statistical significance among three or more groups. A linear regression model was used to test the correlation between two variables. A Fisher’s exact test was used to analyze contingency table data. Statistical calculations were performed with GraphPad Prism Software, Version 7 or 9. Differences with *p*-values of less than 0.05 were considered significant. Statistically significant comparisons in each figure are indicated with asterisks, *p < 0.05, **p < 0.01, ***p < 0.001, and ****p < 0.0001. Data are presented as mean ± SEM.

### Reporting summary

Further information on research design is available in the Nature Portfolio Reporting Summary linked to this article.

### Supplementary information

Supplementary Information

Peer Review File

Reporting Summary

### Source data

Source data

## Data Availability

Source data are provided in the paper. Any additional information needed to reanalyze the data reported in this paper is available from the lead contacts upon request. Source data are provided in this paper.
