# Supplementary material for: α-Synuclein aggregates amplified from patient-derived Lewy bodies recapitulate Lewy body diseases in mice
Source: Nat Commun. 2023 Oct 28;14:6892. doi: 10.1038/s41467-023-42705-5 (PMC10613245; doi:10.1038/s41467-023-42705-5)
Supplement: Supplementary file 1 — Supplementary Information [file 41467_2023_42705_MOESM1_ESM.pdf]

α-Synuclein aggregates amplified from patient-derived Lewy bodies recapitulate Lewy body diseases in mice

Norihito Uemura[1,*], Nicholas P. Marotta[1], Jahan Ara[1], Emily S. Meymand[1], Bin Zhang[1], Hiroshi Kameda[2], Masato Koike[2], Kelvin C. Luk[1], John Q. Trojanowski[1], Virginia M.-Y. Lee[1,*]

*Correspondence: nuemura@kuhp.kyoto-u.ac.jp, vmylee@upenn.edu

Affiliations

[1]Department of Pathology and Laboratory Medicine, Institute on Aging and Center for Neurodegenerative Disease Research, University of Pennsylvania School of Medicine, Philadelphia, Pennsylvania, 19104-2676, USA

[2]Department of Cell Biology and Neuroscience, Juntendo University Graduate School of Medicine, Tokyo, Japan

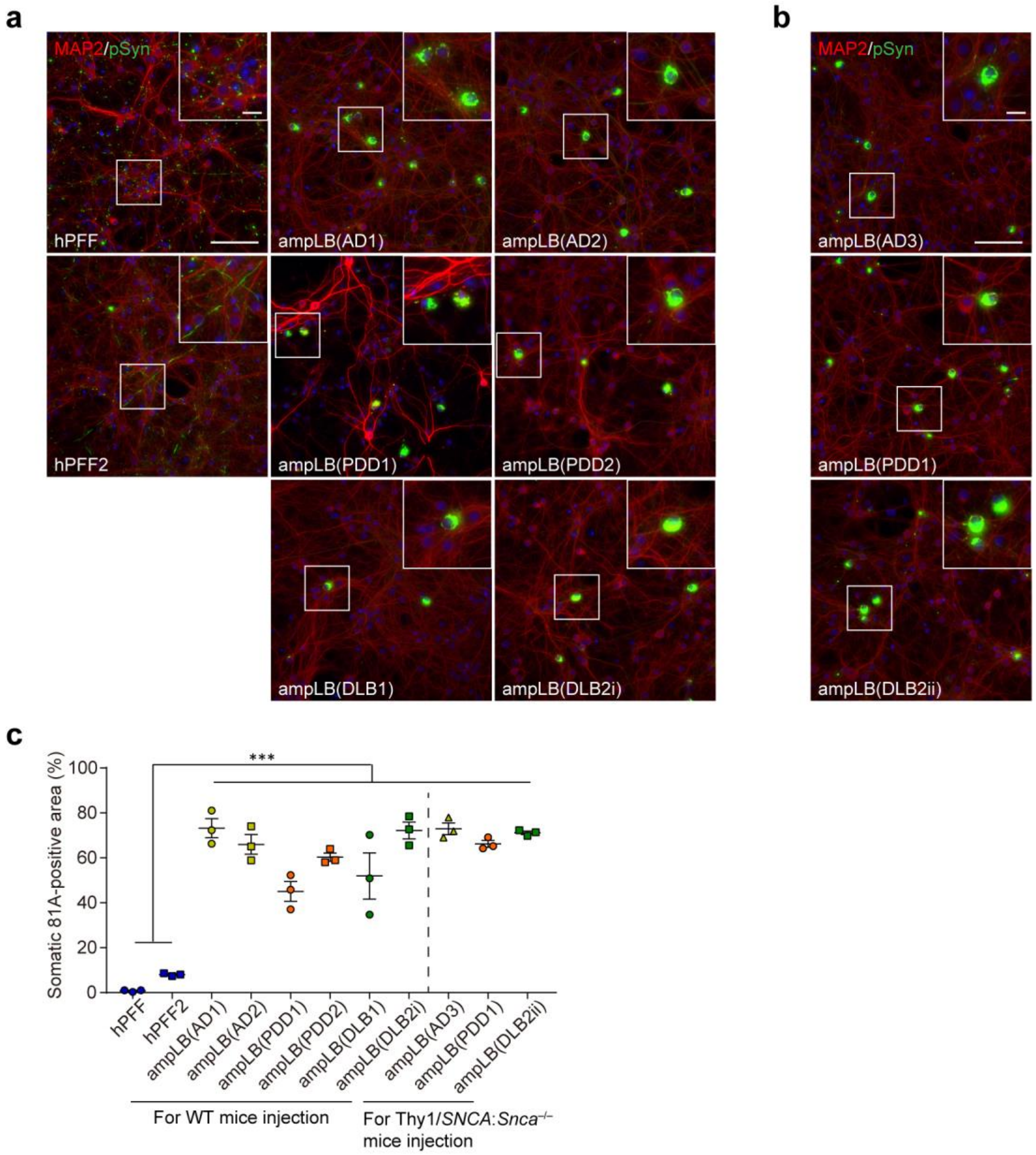

Supplementary Fig. 1 Validation of LB-αSyn amplification in mouse primary neurons. a, b Mouse primary hippocampal neurons treated with hPFF and ampLB preparations. Immunocytochemistry with anti-MAP2 and pSyn (81A) antibodies. Multiple hPFF batches, hPFF used for mouse brain injection and another hPFF batch (hPFF2) were tested. AmpLB preparations used for WT mice injection (**a**) and Thy1:*SNCA*/*Snca*$^{-/-}$ mice injection (**b**) were tested. Scale bars 100 µm, 20 µm (inset). **c** Percent of total pSyn-positive pathology in somatic

inclusions (n = 3 independent wells per group). One-way ANOVA with a Tukey's post-hoc test was performed; hPFF vs. ampLB(AD1), ampLB(AD2), ampLB(PDD1), ampLB(PDD2), ampLB(DLB1), ampLB(DLB2i), ampLB(AD3), ampLB(PDD1), ampLB(DLB2ii) $p < 0.0001$; hPFF2 vs. ampLB(AD1), ampLB(AD2), ampLB(PDD2), ampLB(DLB1), ampLB(DLB2i), ampLB(AD3), ampLB(PDD1), ampLB(DLB2ii) $p < 0.0001$; hPFF2 vs. ampLB(PDD1) $p = 0.001$). Data are represented as mean ± SEM. Source data are provided as a Source Data file.

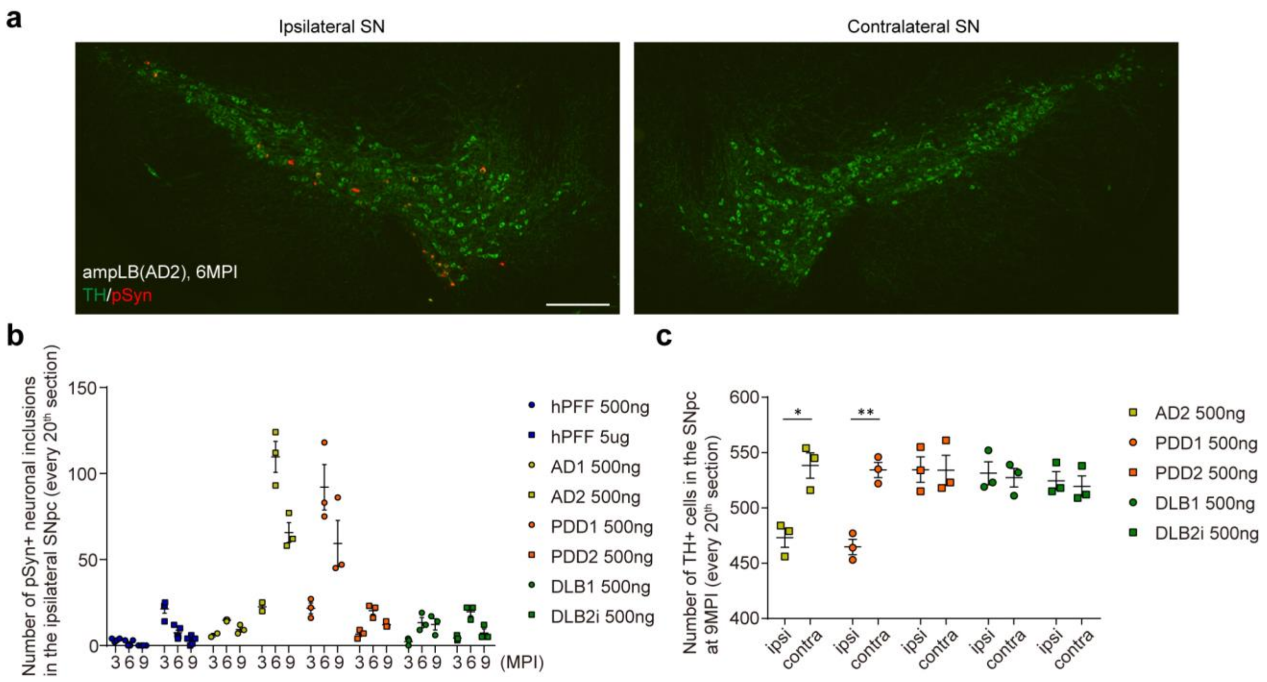

Supplementary Fig. 2 αSyn pathology in the SNpc of ampLB-injected WT mice. a Double immunofluorescence for tyrosine hydroxylase (TH, green) and pSyn (EP1536Y, red) in the SN of ampLB-injected mice at 6MPI. Scale bar 200 µm. **b** Number of pSyn-positive neuronal inclusions in the SNpc (hPFF 500 ng, n = 4; hPFF 5µg, n = 4, 5, 5; AD1, AD2 500 ng, n = 2, 3, 3; PDD1, PDD2, DLB1, DLB2i 500 ng, n = 3 biologically independent samples). **c** Number of TH-positive cells in the ipsilateral (ipsi) and contralateral (contra) SNpc at 9MPI (n = 3 biologically independent samples per group). A two-tailed unpaired Student's *t*-test was performed between the ipsilateral and contralateral sides for each group (AD2 500 ng p = 0.0104; PDD1 500 ng p = 0.0021). Data are represented as mean ± SEM. Source data are provided as a Source Data file.

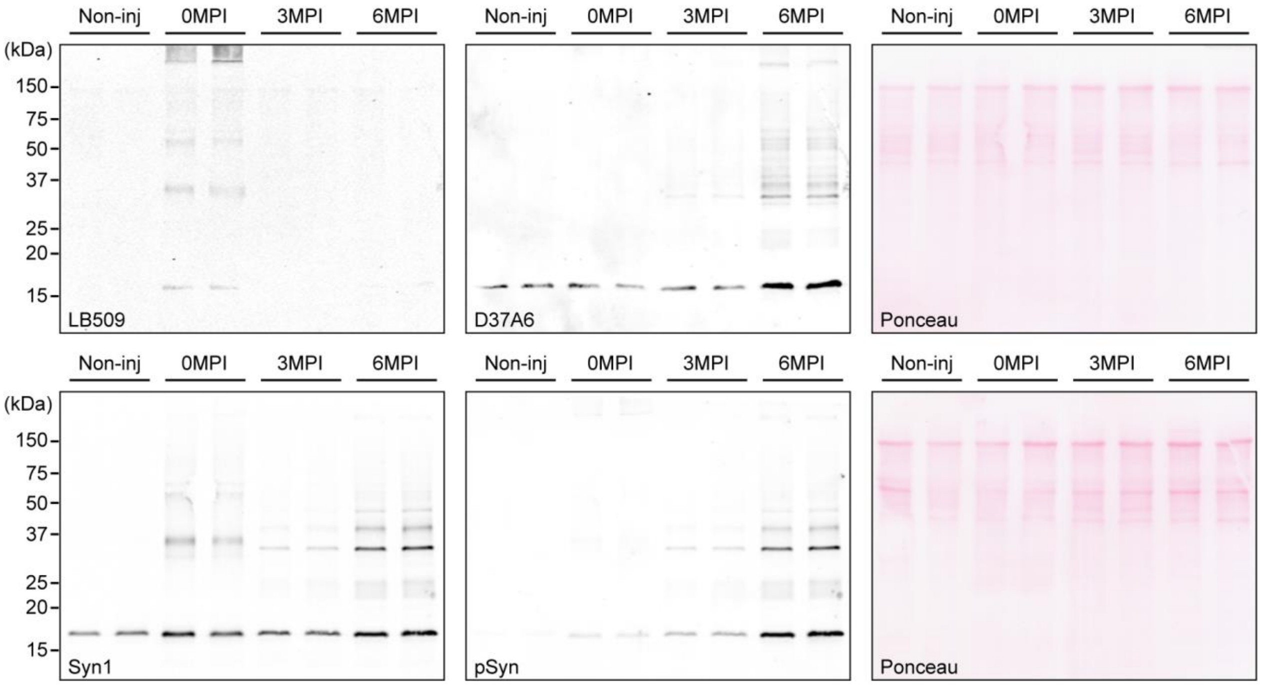

Supplementary Fig. 3 Biochemical analysis of ampLB-injected WT mouse brains. Western blot analysis of sarkosyl-insoluble brain lysates of non-injected mice (Non-inj), ampLB (DLB2i)-injected mice at 0MPI (just after injection), 3MPI, and 6MPI. Immunoblots with anti-αSyn antibodies LB509 (human αSyn), D37A6 (mouse αSyn), and Syn1 (human + mouse αSyn), and an anti-pSyn antibody EP1536Y. Images are representative of two independent experiments. Source data are provided as a Source Data file.

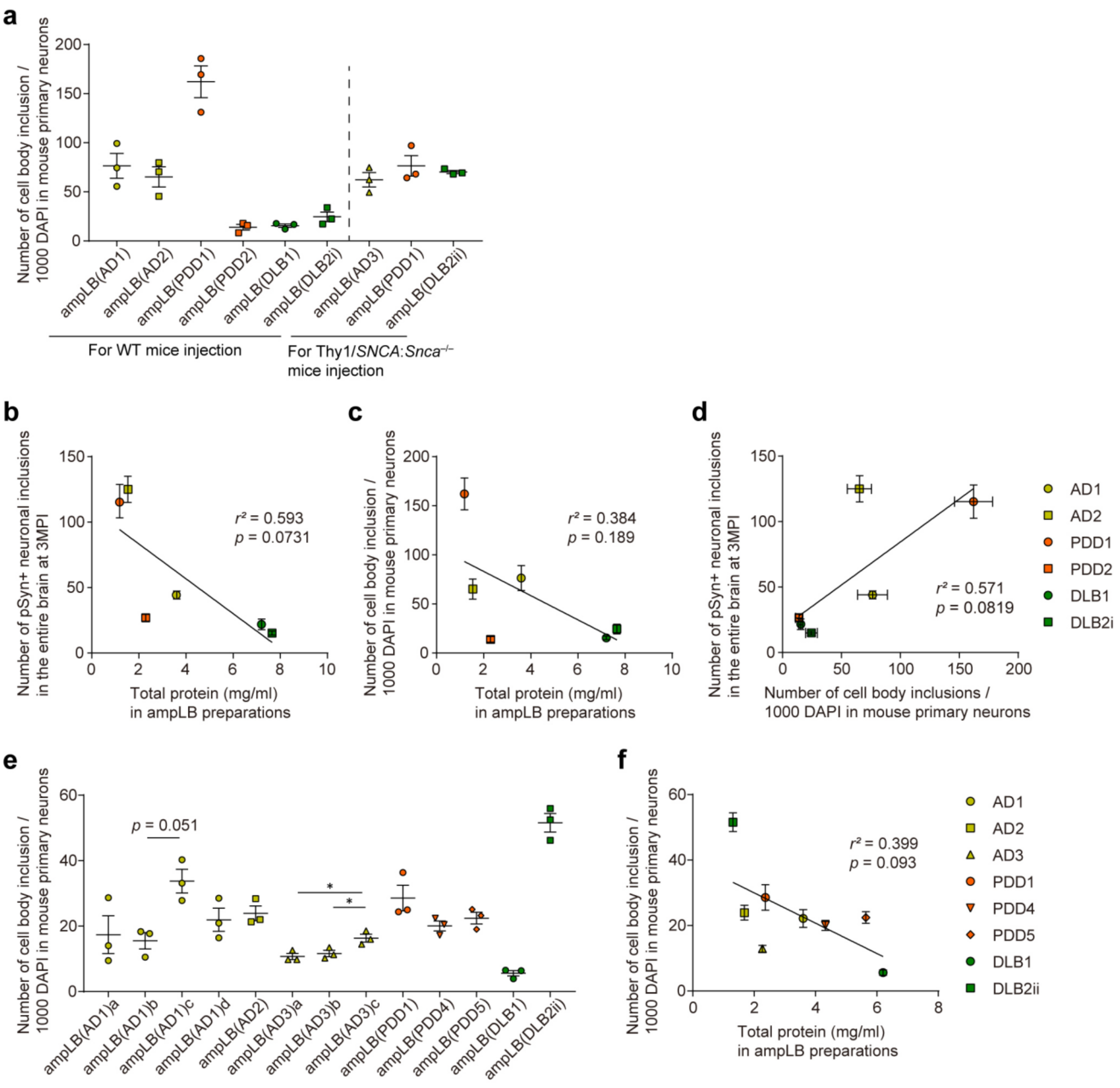

Supplementary Fig. 4 Correlation among *in vivo* and *in vitro* seeding activity and total protein of ampLB preparations. a Number of pSyn-positive cell body inclusions per 1,000 DAPI-positive nuclei in mouse primary hippocampal neurons treated with ampLB in Fig. S1 (n = 3 independent wells per group). **b** Correlation between number of pSyn-positive neuronal inclusions in the entire brains of ampLB-injected mice at 3MPI (AD1, AD2, n = 2; PDD1, PDD2, DLB1, DLB2i, n = 3 biologically independent samples) and total protein in ampLB preparations

(r^2 = 0.593, p = 0.0731). **c** Correlation between number of pSyn-positive cell body inclusions per 1,000 DAPI-positive nuclei in mouse primary hippocampal neurons treated with ampLB (n = 3 independent wells per group) and total protein in ampLB preparations (r^2 = 0.384, p = 0.189). **d** Correlation between number of pSyn-positive neuronal inclusions in the entire brains of ampLB-injected mice at 3MPI and number of pSyn-positive cell body inclusions per 1,000 DAPI-positive nuclei in mouse primary hippocampal neurons treated with ampLB (r^2 = 0.572, p = 0.0819). **e** Number of pSyn-positive cell body inclusions per 1,000 DAPI-positive nuclei in mouse primary hippocampal neurons treated with an independent batch of ampLB preparations (n = 3 independent wells per group). The ampLB(AD1)a–d and ampLB(AD3)a–c preparations were generated from the same brain lysates. One-way ANOVA with a Tukey's post-hoc test was performed among the ampLB(AD1) and ampLB(AD3) preparations (ampLB(AD1)b vs. ampLB(AD1)c p = 0.0508; ampLB(AD3)a, ampLB(AD3)b vs. ampLB(AD3)c p = 0.0206, 0.0428). **f** Correlation between number of pSyn-positive cell body inclusions per 1,000 DAPI-positive nuclei in mouse primary hippocampal neurons treated with ampLB (AD1, n = 12; AD3, n = 9; AD2, PDD1, PDD4, PDD5, DLB1, DLB2ii, n = 3 independent wells) and total protein in ampLB preparations (r^2 = 0.399, p = 0.093). Data are represented as mean ± SEM. Source data are provided as a Source Data file.

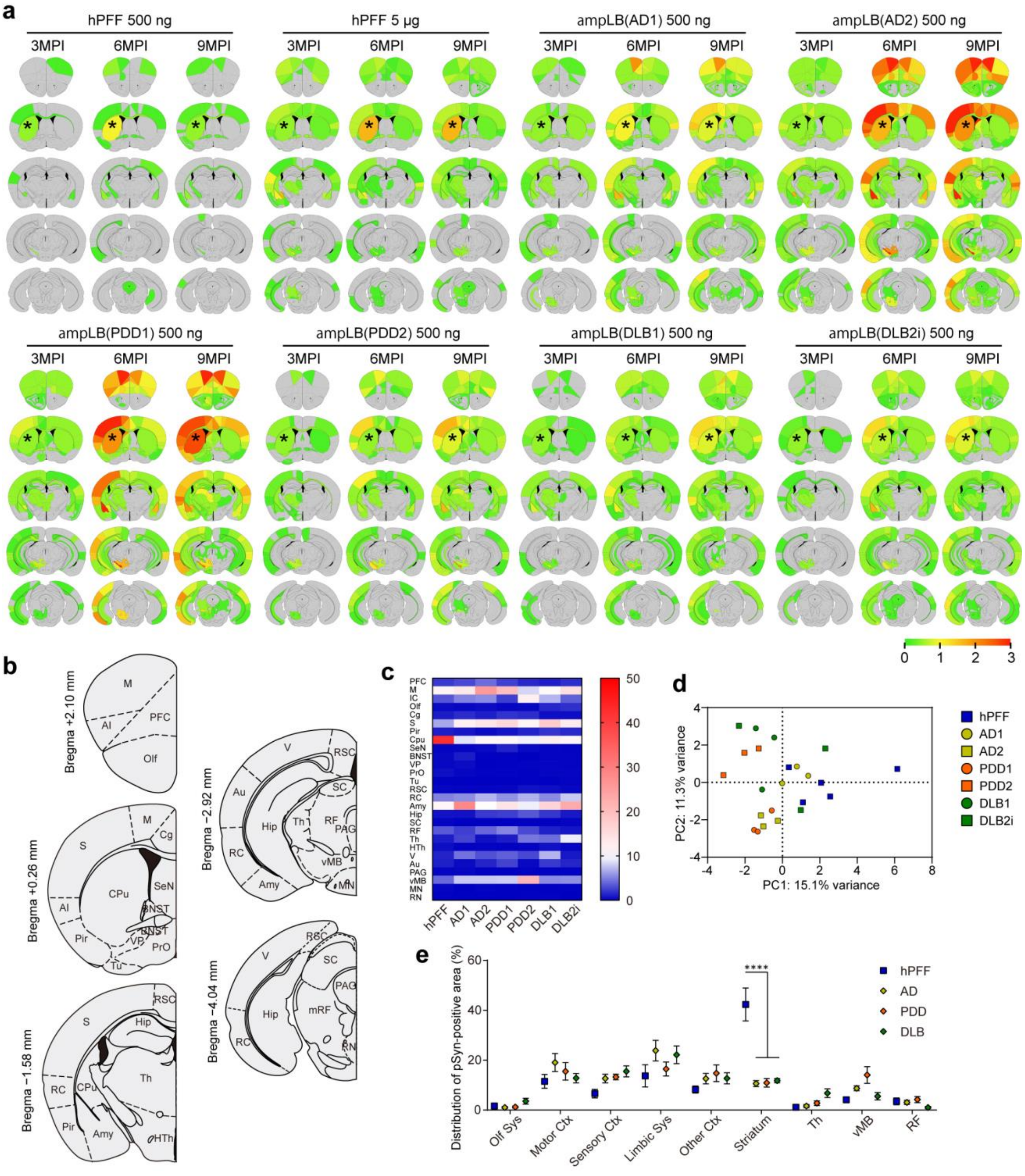

Supplementary Fig. 5 Differences in distribution of αSyn pathology between hPFF and ampLB in WT mice. a Heat map colors represent extent of pSyn-positive pathology. **b** Classification of brain regions for quantitative analyses of pSyn-positive area. **c** Heat map colors

represent proportion of pSyn-positive area in each brain region shown in Fig. 5b at 6MPI. **d** Primary component analysis of the distribution of pSyn-positive pathology at 6MPI (hPFF, n = 5; AD1, AD2, PDD1, PDD2, DLB1, DLB2, n = 3 biologically independent samples). **e** Distribution of pSyn-positive pathology in the brain systems (hPFF, n = 5; AD, PDD, DLB, n = 6 biologically independent samples). Olf Sys, olfactory system; Motor Ctx, motor cortex; Sensory Ctx, sensory cortex; Limbic Sys, limbic system; Th, thalamus; vMB, ventral midbrain; RF, reticular formation. Two-way ANOVA with a Sidak's post-hoc test was performed (interaction $p < 0.0001$, hPFF vs. AD, PDD, DLB $p < 0.0001$). Data are represented as mean ± SEM. Source data are provided as a Source Data file.

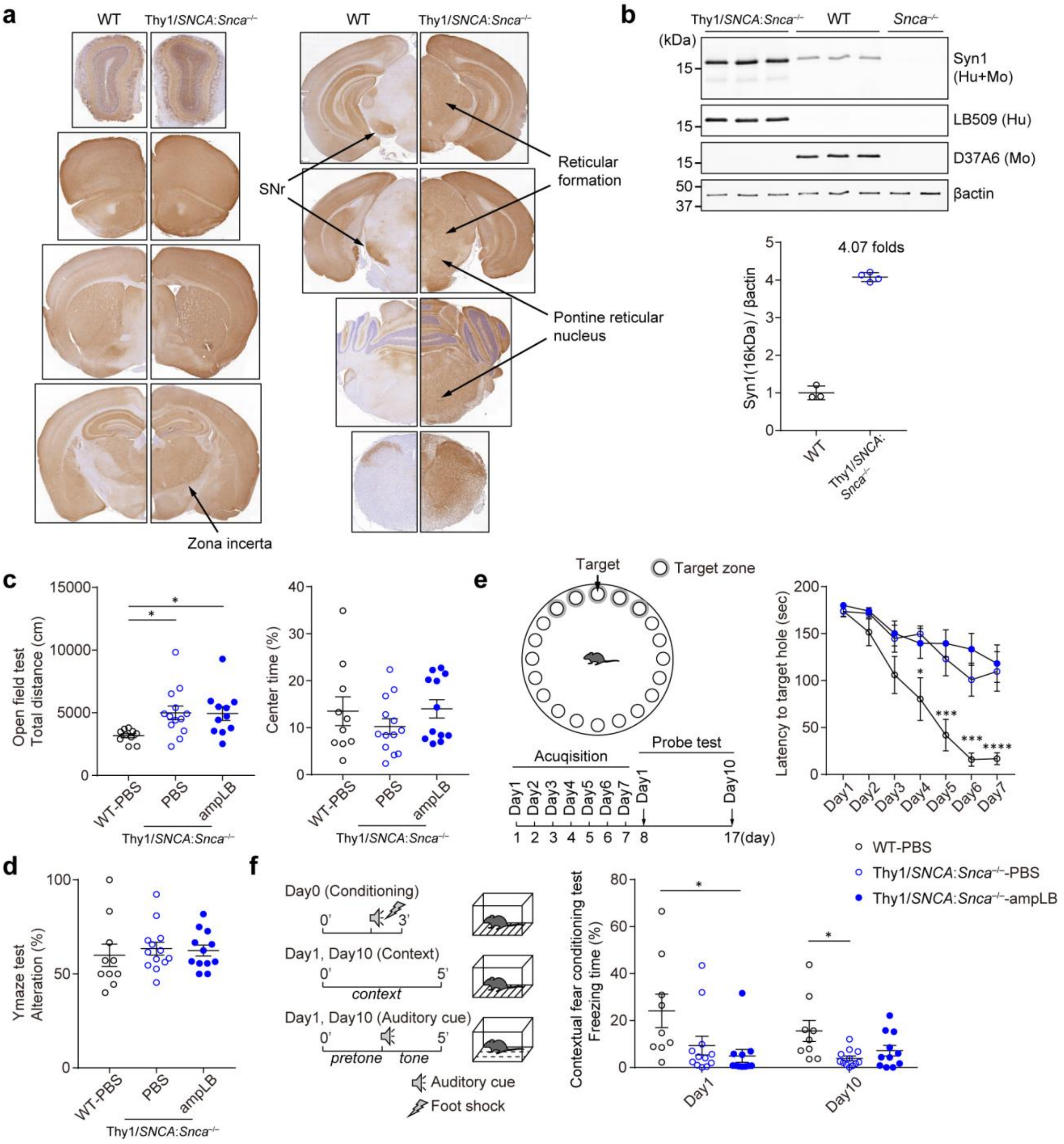

Supplementary Fig. 6 Modeling LBDs in Thy1:*SNCA*/*Snca*$^{-/-}$ mice. a Immunohistochemistry with an anti-αSyn antibody (Syn1). SNr, substantia nigra reticulata. **b** Upper panel: Western blot analysis of Triton X-soluble brain lysates of Thy1:*SNCA*/*Snca*$^{-/-}$ mice, WT mice, and *Snca*$^{-/-}$ mice with anti-αSyn (Syn1 [human and mouse αSyn], LB509 [human αSyn], and D37A6 [mouse αSyn]) and βactin antibodies. Lower panel: quantification of Syn1 (16kDa) bands normalized by

βactin bands (WT, n = 3; Thy1:*SNCA*/*Snca*$^{-/-}$, n = 4 biologically independent samples). **c** Open field test at 7MPI (WT-PBS, n = 10; Thy1:*SNCA*/*Snca*$^{-/-}$-PBS, n = 13; Thy1:*SNCA*/*Snca*$^{-/-}$-ampLB, n = 11 biologically independent samples). Left panel: Total distance traveled. One-way ANOVA with a Tukey's post-hoc test was performed (WT-PBS vs. Thy1:*SNCA*/*Snca*$^{-/-}$-PBS, Thy1:*SNCA*/*Snca*$^{-/-}$-ampLB p = 0.0291, 0.0434). Right panel: Time spent in center area. **d** Y maze test at 7MPI (WT-PBS, n = 10; Thy1:*SNCA*/*Snca*$^{-/-}$-PBS, n = 13; Thy1:*SNCA*/*Snca*$^{-/-}$-ampLB, n = 11 biologically independent samples). **e** Barnes maze test at 7MPI (WT-PBS, n = 10; Thy1:*SNCA*/*Snca*$^{-/-}$-PBS, n = 12; Thy1:*SNCA*/*Snca*$^{-/-}$-ampLB, n = 11 biologically independent samples). Left panel: Schematic representation of experimental design. Right panel: Latency to target hole in acquisition trials. Two-way repeated measures ANOVA with a Tukey's post-hoc test was performed for day (interaction $p < 0.0001$, Day4: WT-PBS vs. Thy1:*SNCA*/*Snca*$^{-/-}$-PBS, Thy1:*SNCA*/*Snca*$^{-/-}$-ampLB p = 0.0021, 0.0123; Day5: WT-PBS vs. Thy1:*SNCA*/*Snca*$^{-/-}$-PBS, Thy1:*SNCA*/*Snca*$^{-/-}$-ampLB p = 0.0002, < 0.0001; Day6: WT-PBS vs. Thy1:*SNCA*/*Snca*$^{-/-}$-PBS, Thy1:*SNCA*/*Snca*$^{-/-}$-ampLB p = 0.0001, < 0.0001; Day7: WT-PBS vs. Thy1:*SNCA*/*Snca*$^{-/-}$-PBS, Thy1:*SNCA*/*Snca*$^{-/-}$-ampLB $p < 0.0001$). **f** Fear conditioning test at 8MPI (WT-PBS, n = 9; Thy1:*SNCA*/*Snca*$^{-/-}$-PBS, n = 12; Thy1:*SNCA*/*Snca*$^{-/-}$-ampLB, n = 11 biologically independent samples). Left panel: Schematic representation of experimental design. Right panel: Freezing time in a contextual fear conditioning test. One-way ANOVA with a Tukey's post-hoc test was performed (Day1: WT-PBS vs. Thy1:*SNCA*/*Snca*$^{-/-}$-ampLB p = 0.0220; Day10: WT-PBS vs. Thy1:*SNCA*/*Snca*$^{-/-}$-PBS p = 0.0118). Source data are provided as a Source Data file.

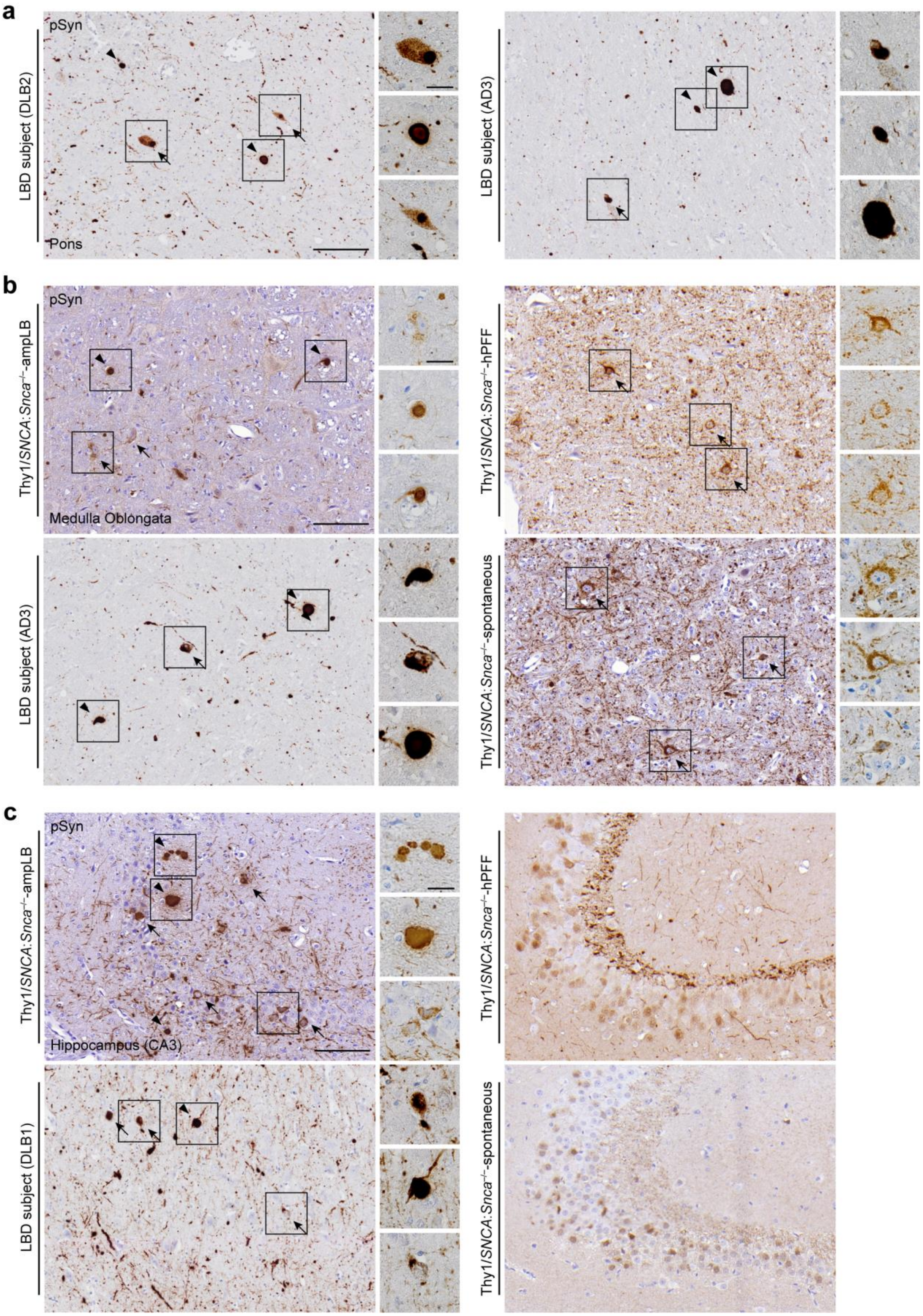
a
pSyn
LBD subject (DLB2)
Pons
LBD subject (AD3)
b
pSyn
Thy1/SNCA:Snca−/−-ampLB
Medulla Oblongata
Thy1/SNCA:Snca−/−-hPFF
LBD subject (AD3)
Thy1/SNCA:Snca−/−-spontaneous
c
pSyn
Thy1/SNCA:Snca−/−-ampLB
Hippocampus (CA3)
Thy1/SNCA:Snca−/−-hPFF
LBD subject (DLB1)
Thy1/SNCA:Snca−/−-spontaneous

Supplementary Fig. 7 Similarities and differences in pathological characteristics among Thy1:*SNCA*/*Snca*$^{-/-}$ mouse models and LBD subjects. Immunohistochemistry with an anti-pSyn antibody (EP1536Y). **a** Pons pathology of the LBD subjects used for brain extraction. **b, c** Medulla oblongata and hippocampus (CA3) pathology of Thy1:*SNCA*/*Snca*$^{-/-}$ mouse models and LBD subjects. Arrows and arrowheads indicate pSyn-positive neuronal inclusions and axonal swelling-like structures, respectively. Scale bars 100 µm, 20 µm (inset).

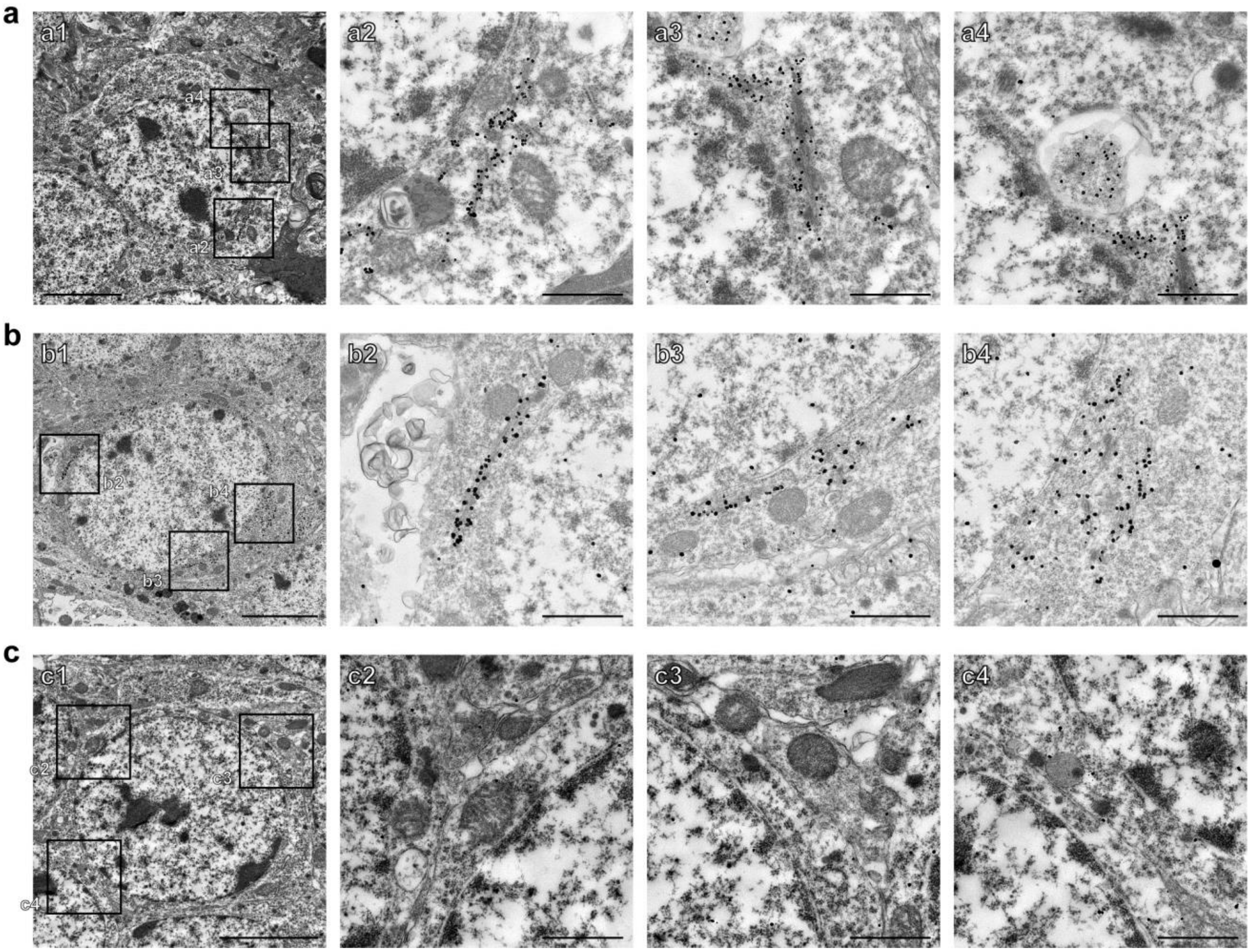

Supplementary Fig. 8 Immunoelectron microscopy images of neuronal inclusions in ampLB- and hPFF-injected Thy1:*SNCA*/*Snca*$^{-/-}$ mice. a–c Immunoelectron micrograph of neurons in the DG with immunogold-labeled pSyn (EP1536Y). **a** pSyn-positive neuron in ampLB-injected Thy1:*SNCA*/*Snca*$^{-/-}$ mice. **b** pSyn-positive neuron in hPFF-injected Thy1:*SNCA*/*Snca*$^{-/-}$ mice. **c** pSyn-negative neuron in ampLB-injected Thy1:*SNCA*/*Snca*$^{-/-}$ mice. Bundles of pSyn-positive filamentous structures in the cytosol (**a2**, **a3**, and **b2–4**) and pSyn-positive amorphous structures in an autophagosome-like vacuole (**a4**). Scale bars 5 µm (**a1**, **b1**, and **c1**) and 1 µm (**a2–4**, **b2–4**, **c2–4**).

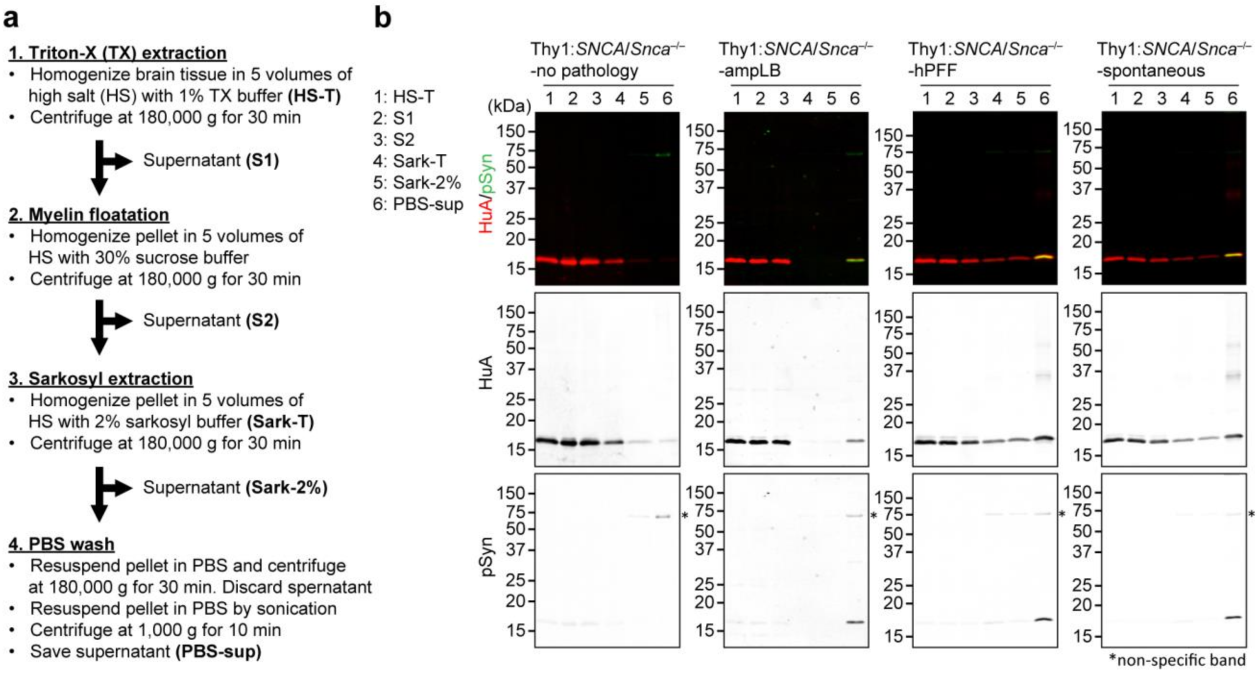

Supplementary Fig. 9 Biochemical extraction of pathological αSyn from Thy1:*SNCA*/*Snca*$^{–/–}$ mouse brains. a Brief description of the mouse brain extraction protocol. **b** Western blot analysis of brain lysates of Thy1:*SNCA*/*Snca*$^{–/–}$ mice without αSyn pathology (Thy1:*SNCA*/*Snca*$^{–/–}$-no pathology), ampLB-injected Thy1:*SNCA*/*Snca*$^{–/–}$ mice (Thy1:*SNCA*/*Snca*$^{–/–}$-ampLB), hPFF-injected Thy1:*SNCA*/*Snca*$^{–/–}$ mice (Thy1:*SNCA*/*Snca*$^{–/–}$-hPFF), and Thy1:*SNCA*/*Snca*$^{–/–}$ mice with spontaneous αSyn pathology (Thy1:*SNCA*/*Snca*$^{–/–}$-spontaneous). Samples containing 10 µg of total protein was loaded to each lane. Immunoblots with anti-human αSyn (HuA) and pSyn (81A) antibodies. Images are representative of three independent experiments. Source data are provided as a Source Data file.

Supplementary Table 1. Clinical information. Clinical information of the cases used for the extraction of pathological αSyn and immunohistochemistry.

**For extraction of pathological αSyn**			
**Case**	**Age of Disease Onset**	**Age at Death**	**PMI (h)**
AD1	68	74	19
AD2	64	72	8
AD3	59	70	18
PDD1	60	72	19
PDD2	72	79	6
PDD3	42	59	10
PDD4	44	73	30
PDD5	53	75	6
DLB1	78	83	9
DLB2	53	63	9
Control	-	62	16

**For immunohistochemistry shown in Fig. 6**			
**Case**	**Age of Disease Onset**	**Age at Death**	**PMI (h)**
LBD	59	76	18.5

Supplementary Table 2. Biochemical information for sarkosyl-insoluble fractions of LBD brain extractions.

Case	Brain region used	Total protein (μg/μl)	αSyn (ng/μl)	Used for
AD1	Middle Frontal Gyrus	14.6	43	AmpLB injection into WT mice
AD2	Middle Frontal Gyrus	8.5	58	AmpLB injection into WT mice
AD3	Middle Frontal Gyrus	13.5	144	AmpLB injection into Thy1:*SNCA*/*Snca*$^{-/-}$ mice
PDD1	Middle Frontal Gyrus	9.3	95	AmpLB injection into WT and Thy1:*SNCA*/*Snca*$^{-/-}$ mice
PDD2	Middle Frontal Gyrus	7.6	36	AmpLB injection into WT mice
PDD3	Middle Frontal Gyrus	17.5	68	Brain lysate injection into WT mice
PDD4	Middle Frontal Gyrus	24	20	Transduction into mouse primary hippocampal neurons
PDD5	Middle Frontal Gyrus	17.5	19	Transduction into mouse primary hippocampal neurons
DLB1	Middle Frontal Gyrus	7.2	12	AmpLB injection into WT mice
DLB2i	Middle Frontal Gyrus	14.9	20	AmpLB injection into WT mice
DLB2ii	Middle Frontal Gyrus	8.8	140	AmpLB injection into Thy1:*SNCA*/*Snca*$^{-/-}$ mice
Control	Middle Frontal Gyrus	4.8	N.D.	Brain lysate injection into WT mice

N.D., not determined.

Supplementary Table 3. Biochemical information for sarkosyl-insoluble fractions of Thy1/SynKO mouse brain extractions.

Mice	Total protein (µg/µl)	αSyn (ng/µl)
Thy1:*SNCA*/*Snca*$^{-/-}$-ampLB(PDD1)-1	5.11	15.0
Thy1:*SNCA*/*Snca*$^{-/-}$-ampLB(PDD1)-2	3.26	6.32
Thy1:*SNCA*/*Snca*$^{-/-}$-ampLB(DLB2ii)-1	3.75	10.2
Thy1:*SNCA*/*Snca*$^{-/-}$-ampLB(DLB2ii)-2	5.55	15.85
Thy1:*SNCA*/*Snca*$^{-/-}$-hPFF-1	4.21	40.0
Thy1:*SNCA*/*Snca*$^{-/-}$-hPFF-2	2.49	65.0
Thy1:*SNCA*/*Snca*$^{-/-}$-spontaneous-1	1.68	26.8
Thy1:*SNCA*/*Snca*$^{-/-}$-spontaneous-2	4.25	53.5
Thy1:*SNCA*/*Snca*$^{-/-}$-no pathology	4.18	1.57
