# Supplementary material for: α-Synuclein aggregates amplified from patient-derived Lewy bodies recapitulate Lewy body diseases in mice
Source: Nat Commun. 2023 Oct 28;14:6892. doi: 10.1038/s41467-023-42705-5 (PMC10613245; doi:10.1038/s41467-023-42705-5)
Supplement: Supplementary file 3 — Reporting Summary [file 41467_2023_42705_MOESM3_ESM.pdf]

nature portfolio

Corresponding author(s): Norihito Uemura, Virginia M.-Y. Lee

Last updated by author(s): Oct 17, 2023

Reporting Summary

Nature Portfolio wishes to improve the reproducibility of the work that we publish. This form provides structure for consistency and transparency in reporting. For further information on Nature Portfolio policies, see our Editorial Policies and the Editorial Policy Checklist.

Statistics

For all statistical analyses, confirm that the following items are present in the figure legend, table legend, main text, or Methods section.

n/a	Confirmed	
☐	☒	The exact sample size (*n*) for each experimental group/condition, given as a discrete number and unit of measurement
☐	☒	A statement on whether measurements were taken from distinct samples or whether the same sample was measured repeatedly
☐	☒	The statistical test(s) used AND whether they are one- or two-sided<br>*Only common tests should be described solely by name; describe more complex techniques in the Methods section.*
☒	☐	A description of all covariates tested
☐	☒	A description of any assumptions or corrections, such as tests of normality and adjustment for multiple comparisons
☐	☒	A full description of the statistical parameters including central tendency (e.g. means) or other basic estimates (e.g. regression coefficient) AND variation (e.g. standard deviation) or associated estimates of uncertainty (e.g. confidence intervals)
☐	☒	For null hypothesis testing, the test statistic (e.g. *F*, *t*, *r*) with confidence intervals, effect sizes, degrees of freedom and *P* value noted<br>*Give P values as exact values whenever suitable.*
☒	☐	For Bayesian analysis, information on the choice of priors and Markov chain Monte Carlo settings
☒	☐	For hierarchical and complex designs, identification of the appropriate level for tests and full reporting of outcomes
☒	☐	Estimates of effect sizes (e.g. Cohen's *d*, Pearson's *r*), indicating how they were calculated

Our web collection on statistics for biologists contains articles on many of the points above.

Software and code

Policy information about availability of computer code

Data collection	Image Studio was used to collect western blot data. IN Cell Analyzer 2200 software was used to image stained primary neurons. Molecular Devices Spectramax M5 plate reader software was used to read ELISA and BCA assay plates. Pannoramic 250 software was used to collect immunohistochemical images. Eclipse Ni microscope and TCS SP8 WLL Confocal with STED 3X software was used to collect immunofluorescent images. EthoVision XT 15 and fear conditioning test device software were used to collect behavioral data.
Data analysis	ImageJ was used to quantify western blot data. IN Cell Analyzer 2200 software was used to analyze primary neuron images. Cell Profiler was used to quantify staining area in primary neurons. QuPath was used to quantify staining area in immunohistochemical images. GraphPad Prism 7 or 9 was used to generate standard curves and for statistical analysis.

For manuscripts utilizing custom algorithms or software that are central to the research but not yet described in published literature, software must be made available to editors and reviewers. We strongly encourage code deposition in a community repository (e.g. GitHub). See the Nature Portfolio guidelines for submitting code & software for further information.

Data

Policy information about availability of data

All manuscripts must include a data availability statement. This statement should provide the following information, where applicable:

- Accession codes, unique identifiers, or web links for publicly available datasets
- A description of any restrictions on data availability
- For clinical datasets or third party data, please ensure that the statement adheres to our policy

All raw data and uncropped blots are provided in a Source Data file.

Research involving human participants, their data, or biological material

Policy information about studies with human participants or human data. See also policy information about sex, gender (identity/presentation), and sexual orientation and race, ethnicity and racism.

Reporting on sex and gender	Sex information of human subjects was described in Table S1.
Reporting on race, ethnicity, or other socially relevant groupings	Race information of human subjects was described in Table S1.
Population characteristics	This study used postmortem tissue. The clinical diagnosis, pathological diagnosis, age of disease onset, age at death, and postmortem interval were described in Table S1.
Recruitment	This study used postmortem tissue from donors who underwent autopsy at CNDR between 2002 and 2018.
Ethics oversight	The use of postmortem tissue in this study was approved by the Institutional Review Boards of University of Pennsylvania.

Note that full information on the approval of the study protocol must also be provided in the manuscript.

Field-specific reporting

Please select the one below that is the best fit for your research. If you are not sure, read the appropriate sections before making your selection.

☒ Life sciences ☐ Behavioural & social sciences ☐ Ecological, evolutionary & environmental sciences

For a reference copy of the document with all sections, see nature.com/documents/nr-reporting-summary-flat.pdf

Life sciences study design

All studies must disclose on these points even when the disclosure is negative.

Sample size	No statistical methods were used to predetermine sample sizes but our sample sizes are similar to those reported in previous publications cited in the manuscript (Peng C et al., Nature. 2018; Marotta et al., Acta Neuropahtol Commun. 2021).
Data exclusions	In behavioral analyses, samples that encountered technical problems were removed from the data analyses.
Replication	All the biological replicates supporting reproducibility are indicated in each figure legend.
Randomization	For all the animal and cultured cell studies, samples were randomly assigned to each experimental group.
Blinding	The investigators were blinded to group allocation for mouse behavioral analyses and quantitative analyses on pathological images. The investigators were not blinded to quantitative analyses on cultured cells because they were automatically conducted with a software.

Reporting for specific materials, systems and methods

We require information from authors about some types of materials, experimental systems and methods used in many studies. Here, indicate whether each material, system or method listed is relevant to your study. If you are not sure if a list item applies to your research, read the appropriate section before selecting a response.

Materials & experimental systems

n/a	Involved in the study	
[ ]	[x]	Antibodies
[x]	[ ]	Eukaryotic cell lines
[x]	[ ]	Palaeontology and archaeology
[ ]	[x]	Animals and other organisms
[x]	[ ]	Clinical data
[x]	[ ]	Dual use research of concern
[x]	[ ]	Plants

Methods

n/a	Involved in the study	
[x]	[ ]	ChIP-seq
[x]	[ ]	Flow cytometry
[x]	[ ]	MRI-based neuroimaging

Antibodies

Antibodies used

Mouse anti-α-synuclein monoclonal antibody, CNDR, Syn9027
Rabbit anti-α-synuclein monoclonal antibody, Abcam, MJFR1; Cat#ab138501
Rabbit anti-α-synuclein polyclonal antibody, CNDR, HuA
Mouse anti-pS129 α-synuclein monoclonal antibody, CNDR, 81A
Rabbit anti-MAP2 polyclonal antibody, CNDR, #17028
Mouse anti-α-synuclein monoclonal antibody, CNDR, LB509
Rabbit anti-α-synuclein monoclonal antibody, Cell Signaling, D37A6; Cat#4179
Mouse anti-α-synuclein monoclonal antibody, BD transduction, Syn1; Cat#610787
Rabbit anti-pS129 α-synuclein monoclonal antibody, Abcam, EP1536Y; Cat#ab51253
Mouse anti-pS129 α-synuclein monoclonal antibody, Wako, #64; Cat#015-25191
Mouse anti-TH monoclonal antibody, Sigma-Aldrich, TH-16; Cat#T2928
Mouse anti-NeuN monoclonal antibody, Sigma-Aldrich, A60; Cat#MAB377
Rat anti-GFAP monoclonal antibody, CNDR, 2.2B10
Rabbit anti-Iba1 polyclonal antibody, Wako, Cat#019-19741
Rat anti-phosphorylated neurofilament monoclonal antibody, CNDR, TA51
Biotinylated horse anti-mouse IgG (H+L) antibody, Vector laboratories, Cat#BA-2000
Biotinylated horse anti-rabbit IgG (H+L) antibody, Vector laboratories, Cat#BA-1000
Biotinylated horse anti-rat IgG (H+L) antibody, Vector laboratories, Cat#BA-4000
Alexa Fluor 488 conjugated goat anti-mouse IgG (H+L) Cross-Adsorbed Secondary Antibody, Invitrogen, Cat#A11029
Alexa Fluor 488 conjugated goat anti-rabbit IgG (H+L) Highly Cross-Adsorbed Secondary Antibody, Invitrogen, Cat#A11034
Alexa Fluor 488 conjugated goat anti-rat IgG (H+L) Cross-Adsorbed Secondary Antibody, Invitrogen, Cat#A11006
Alexa Fluor 594 conjugated goat anti-mouse IgG (H+L) Highly Cross-Adsorbed Secondary Antibody, Invitrogen, Cat#A11032
Alexa Fluor 594 conjugated goat anti-rabbit IgG (H+L) Highly Cross-Adsorbed Secondary Antibody, Invitrogen, Cat#A11037

Validation

Anti-α-synuclein antibody Syn9027 was validated previously (Covell DJ et al., Neuropathol Appl Neurobiol. 2017).
Anti-α-synuclein antibody MJFR was verified by Abcam (https://www.abcam.com/alpha-synuclein-antibody-mjfr1-ab138501.html).
Anti-α-synuclein antibody HuA was validated previously (Giasson BI et al., J Neurosci Res. 2000).
Anti-pS129 α-synuclein antibody 81A was validated previously (Waxman EA et al., Acta Neuropathol. 2008).
Anti-MAP2 antibody #17028 was validated previously (Volpicelli-Daley LA et al., Neuron. 2011).
Anti-α-synuclein antibody LB509 was validated previously (Jakes R et al., Neurosci Lett. 1999).
Anti-α-synuclein antibody D37A6 was validated by Abcam (https://www.cellsignal.com/products/primary-antibodies/a-synuclein-d37a6-rabbit-mab/4179).
Anti-α-synuclein antibody Syn1 was validated by BD transduction (https://www.bdbiosciences.com/en-eu/products/reagents/microscopy-imaging-reagents/immunofluorescence-reagents/purified-mouse-anti-synuclein.610787).
Anti-pS129 α-synuclein antibody EP1536Y was validated by Abcam (https://www.abcam.com/alpha-synuclein-phospho-s129-antibody-ep1536y-ab51253.html).
Anti-pS129 α-synuclein antibody #64 was validated by Wako (https://labchem-wako.fujifilm.com/us/product/detail/W01W0101-2648.html).
Anti-TH antibody TH-16 was validated by Sigma-Aldrich (https://www.sigmaaldrich.com/US/en/product/sigma/t2928).
Anti-NeuN antibody A60 was validated by Sigma-Aldrich (https://www.sigmaaldrich.com/US/en/product/mm/mab377?gclid=Cj0KCQiAi8KfBhCuARIsADp-A56mLJL5-dlcWQ9zR8KsSVt4VCA9of5hM5HVa31Dqkl2LXy0PW3qCPkaAjhWEALw_wcB).
Anti-GFAP antibody 2.2B10 was validated previously (Lee VM et al., J Neurochem. 1984).
Anti-Iba1 antibody was validated by Wako (https://labchem-wako.fujifilm.com/us/product/detail/W01W0101-1974.html)
Anti-phosphorylated neurofilament antibody TA51 was validated previously (Lee VM et al., J Neurosci. 1987).

Animals and other research organisms

Policy information about studies involving animals; ARRIVE guidelines recommended for reporting animal research, and Sex and Gender in Research

Laboratory animals

Pregnant CD-1 mice at 2–3 months of age were purchased from Charles River for the isolation of primary neurons. C57BL/6 C3H (B6C3) mice at 2–3 months of age were purchased from Charles River for in vivo experiments. Thy1:SNCA/Snca–/– mice were generated in CNDR by crossing Thy1:SNCA mouse line 61 (Rockenstein E et al., J Neurosci Res. 2002) with Snca–/– mice (Abeliovich A

	et al., Neuron. 2000).
Wild animals	This study did not include any wild animals.
Reporting on sex	The major findings in this study can apply to both sexes. Sex was considered in the study design, and we used only male Thy1:SNCA/Snca–/– mice because the transgene was inserted in the X chromosome (Rockenstein E et al., J Neurosci Res. 2002). Sex-based analyses were not performed because this study did not focus on sex differences in LBDs.
Field-collected samples	This study did not include any samples collected from the field.
Ethics oversight	All animal procedures were approved by the University of Pennsylvania Institutional Animal Care and Use Committee and conformed to the National Institute of Health Guide for Care and Use of Laboratory Animals.

Note that full information on the approval of the study protocol must also be provided in the manuscript.
